# Donor–Acceptor Bridging via Twisted Quasi-Double-Bond: A Facile Design Strategy for Typical NIR-II AIEgen with “Simple” Structure

**DOI:** 10.1021/cbmi.5c00302

**Published:** 2026-04-08

**Authors:** Chunbin Li, Jen-Shyang Ni, Pengfei Zhang, Daoyong Jiang, Jianguo Wang, Kai Li, Shuang-Quan Zang, Ryan T. K. Kwok, Weihong Tan, Ben Zhong Tang

**Affiliations:** † Department of Chemistry, The Hong Kong Branch of Chinese National Engineering Research Center for Tissue Restoration and Reconstruction, State Key Laboratory of Nervous System Disorder, Division of Life Science, and Department of Chemical and Biological Engineering, 58207 The Hong Kong University of Science and Technology, Kowloon, Hong Kong 999077, China; ‡ Guangdong Basic Research Center of Excellence for Aggregate Science, School of Science and Engineering, Shenzhen Institute of Aggregate Science and Technology, 407605 The Chinese University of Hong Kong, Shenzhen (CUHK-Shenzhen), Shenzhen 518172, China; § Guangdong Key Laboratory of Nanomedicine, CAS Key Laboratory of Health Informatics, Shenzhen Bioactive Materials Engineering Lab for Medicine, Institute of Biomedicine and Biotechnology, Shenzhen Institutes of Advanced Technology, Chinese Academy of Sciences, Shenzhen 518055, China; ∥ Department of Biomedical Engineering, Southern University of Science and Technology, Shenzhen, Guangdong 518055, China; ⊥ School of Chemistry and Life Sciences, Guilin Normal University, Guilin 541199, China; # College of Chemistry and Chemical Engineering, College of Biomedical Sciences, Inner Mongolia Key Laboratory of Synthesis and Application of Organic Functional Molecules, 12576 Inner Mongolia University, Hohhot 010021, China; ∇ College of Chemistry and Molecular Engineering, 12636 Zhengzhou University, Science Road No. 100, Zhengzhou 450001, China; ○ Hangzhou Institute of Medicine (HIM), 12381 Chinese Academy of Sciences, Hangzhou, Zhejiang 310022, China

**Keywords:** near-infrared II, aggregation-induced emission, acceptor−donor bridging, fluorescent probes, in vivo imaging

## Abstract

A facile design strategy for NIR-II fluorophores with aggregation-induced emission (AIE) characteristics was presented. This kind of NIR-II AIE luminogen, TSPCI is an example, is by the strong acceptor–donor bridging via a twisted quasi-double-bond (TQDB). Theoretical calculations revealed that the introduction of TQDB was the major factor for quenching of the photoexcited TSPCI molecule in the solution state. TSPCI showed excellent NIR-II imaging performance for in vivo dynamic imaging with high contrast and penetration depth. TSPCI holds great potential as a building block to easily fabricate activatable fluorescent probes with superior responsive ability in the second NIR optical window for in vivo imaging applications.

## Graphical abstract

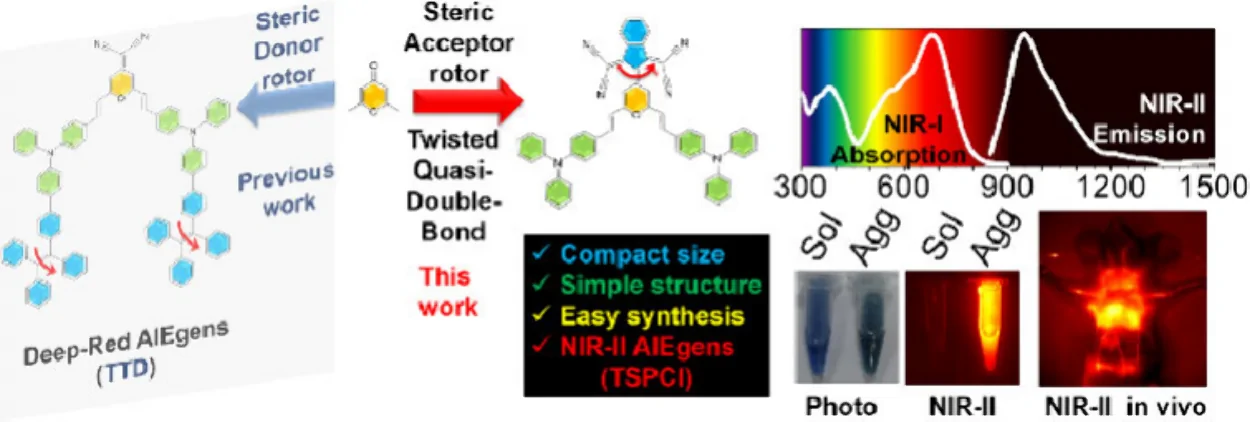

As a rapid, non-invasive, sensitive, and low-cost approach, the near-infrared (NIR) fluorescence imaging technique is highly attractive for direct and real-time visualizing dynamic biological/physiological processes in vivo. These NIR fluorescent images having outstanding temporal and spatial resolution offering useful functional and structural insights in complex biological environments.^1–3^ Particularly, due to minimal photon scattering and negligible tissue autofluorescence located in the spectrum range 1000–1700 nm (the second near-infrared widow, NIR-II), NIR-II fluorescence imaging provides great improvement on the imaging performance with higher image contrast and much greater penetration depth as compared to conventional NIR-I (650–900 nm) fluorescence imaging.^4–9^ Until now, various NIR-II fluorescent probes have been developed for in vivo deep-tissue imaging with high spatial/temporal resolution, such as single-walled carbon nanotubes,^10–13^ quantum dots,^14–22^ noble metal nanoclusters,^23^ rare-earth doped nanoparticles,^24–26^ semiconductor polymer nanoparticles,^27,28^ dye-doped nanoparticles,^29,30^ etc. Regarding clinical translation, small molecular organic fluorescent probes show much greater potential for clinical translation. At present, only two small organic molecules, indocyanine green (ICG)^31^ and methylene blue (MB),^32^ have been approved by the U.S. Food and Drug Administration (FDA) for in vivo clinical NIR imaging. Recent studies further revealed that the emission spectrum of ICG exhibits a tailing peak at the region of NIR-II.^33^ However, the library of NIR-II fluorescent small organic molecules still needs to be expanded.

There are two common strategies for the rational design of NIR emissive fluorophores. The first step is to increase the molecular conjugation length. Various NIR polymethine dyes with elongated π-electron structure have been developed since the 1930s, including IR-26, IR-1048, IR-1061, Flav7, and FD-1080.^34–37^ The major drawback of them is their small Stokes shift, which brings about a high interference from the excitation light. On the other hand, the alternative strategy is judiciously combining the electron donor (D) and acceptor (A). A series of D–A–D structural NIR-II dyes have been designed and synthesized by bridging strong electron withdrawing (e.g., benzobisthiadiazole, BBTD) and electron-donating groups.^38–42^ Compared to traditional polymethine dyes, this D–A–D architecture not only enables tunable emission in the NIR-II region but also affords a significantly larger Stokes shift, which minimizes self-absorption, reduces excitation light interference, and improves imaging signal-to-background ratio (SBR) advantages that are particularly valuable for in vivo imaging applications. However, the complex molecular structures, multiple synthetic steps with low yields, and troublesome purification procedure significantly hinder the widespread use of these NIR-II imaging probes in further preclinical and clinical studies. Therefore, there is still an urgent need to develop a simple and facile synthetic strategy for efficiently NIR-II-emissive fluorophores in biological environments.

Besides, the hydrophobic nature of NIR organic dyes with large conjugated backbones makes them intrinsically able to form aggregates in the hydrophilic bioenvironments, which may lead to severely weakened or quenched fluorescence due to strong π–π stacking (known as aggregation-caused quenching, ACQ). Different from additional functionalization to increase aqueous solubility, the unique aggregation-induced emission (AIE) process offers a straightforward solution to the ACQ problem.^43,44^ The fluorogens with AIE characteristics (AIEgens) show weak or even no emission in a good solvent, where the dispersed molecular species have active intramolecular motions. Upon aggregation in a medium with poor solubility, they are highly emissive due to the restriction of intramolecular motion (RIM) process.^45^ More importantly, environmental/target-responsive fluorescent bioprobes with turn-on behavior could be easily constructed by taking advantage of typical AIE process.^46,47^ Although it is a well-recognized strategy for developing long-wavelength emissive AIEgens by introducing AIE-active units to traditional organic dyes with large π-conjugation, it is still challenging to pursue AIEgens with “simple” structure and NIR excitation/emission.^48,49^

Tetraphenylethylene (TPE) and triphenylamine (TPA) derivatives have been well studied and applied in various fields because of their typical AIE behavior, “simple” chemical structure, and convenient preparation.^50^ Recently fundamental studies have revealed that the torsion of a double bond plays a crucial role in the nonradiative energy decay of TPE derivatives in solution,^51,52^ which inspires us to develop a new kind of D–A–D structural NIR fluorophores with typical AIE behavior by bridging strong electron acceptors and strong electron donors through atwisted double bond. In this work, we intended to utilize commercially available chemicals as starting materials and developed a facile method to obtain a “simple” structural AIEgen, TSPCI, with both NIR absorption and emission, in which 1,3-bis­(dicyanomethylene)­indan (BDCI) was served as an building block of both strong and steric electron-deficient acceptor in optoelectronic materials design.^53,54^ Further imaging performance comparison experiments of as-prepared NIR-II AIEgens in comparison with deep-red/NIR-I AIEgens or the commercial IR-1048 (NIR-II ACQ dye) revealed that TSPCI not only was capable of high-resolution/contrast imaging with deep penetration but also showed great potential as scaffold to easily develop an environment-responsive light-up probe with NIR-II fluorescence for in vivo biological imaging with high contrast.

TSPCI was easily synthesized in just two steps from commercially available starting materials (Scheme 1). BDCI was reacted with 2,6-dimethyl-4-pyrone (**1**) in acetic anhydride at 120 °C to yield the intermediate, SPCI, which was further condensed with 4-(*N*,*N*-diphenylamino)­benzaldehyde (**2**) and 4-dimethylaminocinnamaldehyde (**3**) to obtain TSPCI and MASPCI, respectively (Figure 1a). The intermediate and desirable products were characterized by the standard spectroscopy methods with satisfactory results, as depicted in Figures S2–S7. The UV–vis spectra of TSPCI and MASPCI measured in tetrahydrofuran (THF) solution showed maximal absorption wavelengths at 682 and 723 nm, with relatively high molar extinction coefficients (*ε* up to 6.67 × 10^4^ M^–1^ cm^–1^ for TSPCI and 4.78 × 10^4^ M^–1^ cm^–1^ for MASPCI) (Figure 1B and Figure S8 and Table S1). Notably, TSPCI exhibits a distinct absorption peak at around 400 nm, which primarily originates from the π–π* electronic transition of the conjugated molecular backbone as well as the characteristic absorption of TPA donor. Under 808 nm laser irradiation, TSPCl exhibits negligible fluorescence emission in THF solution (Figure 1B). We then investigated the AIE property of TSPCI by varying the water fractions (*f*_w_) in the THF/water mixtures (Figure 1C). Upon the increase in *f*_w_, a significant fluorescence enhancement was observed clearly. The strongest emission intensity at the maximum emission wavelength reached its maximum value at 90% water content, which was over 10-fold higher than that in pure THF (Figure 1D). In addition, the AIE curve of MASPCl was also measured in THF/water mixtures. When *f*_w_ was less than 50%, the fluorescence intensity gradually decreased with increasing *f*_w_, accompanied by a red shift in the emission wavelength, which was attributed to the twisted intramolecular charge transfer (TICT) effect. When *f*_w_ exceeded 50%, the fluorescence gradually increased, exhibiting AIE characteristics (Figure S9). The enhanced AIE property of TSPCl enables us to further investigate other photophysical properties. According to previous work of the Dai group, NIR-II fluorophores with quantum yield (QY) of 0.3–0.7% could be successfully used to directly visualize in vivo biological processes with high-spatial and temporal resolution due to the reduced scattering, minimum absorption, and negligible autofluorescence in the NIR-II spectral window and optimum high detection sensitivity of the NIR-II InGaAs camera with extremely low readout thermal noise. The QY of TSPCI aggregates was measured to be 0.7% using IR-26 as the reference standard sample (QY_IR‑26_ = 0.5% in 1,2-dichloroethane). Additionally, the reactive oxygen species (ROS) generation capability and photothermal properties of TSPCI were further evaluated. DCFH was utilized as a commercial probe for ROS detection. Upon continuous irradiation with an 808 nm laser, the fluorescence change of DCFH in the presence of TSPCI aggregates was significantly higher than that of DCFH alone, indicating excellent ROS generation capability and suggesting the potential for photodynamic therapy (Figure S10). Notably, TSPCI exhibited a negligible photothermal effect under 808 nm irradiation in both solution and aggregate states (Figure S11). This result indicates that the thermal interference from TSPCI during imaging is minimal, demonstrating its safety for imaging applications.

**Scheme 1:**
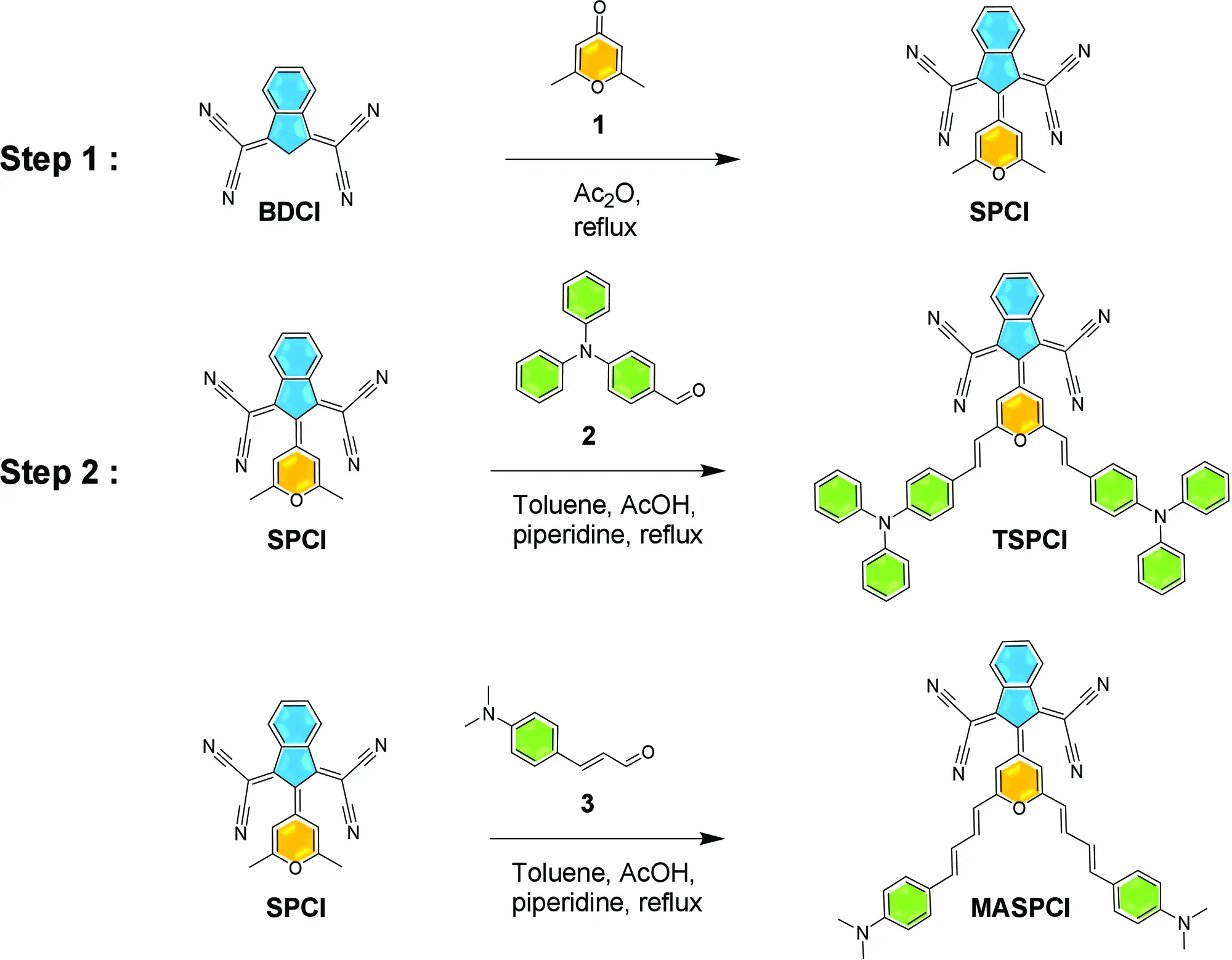
Molecular Structure and Two-Step Synthetic Routes of TSPCI and MASPCI

**Figure 1:**
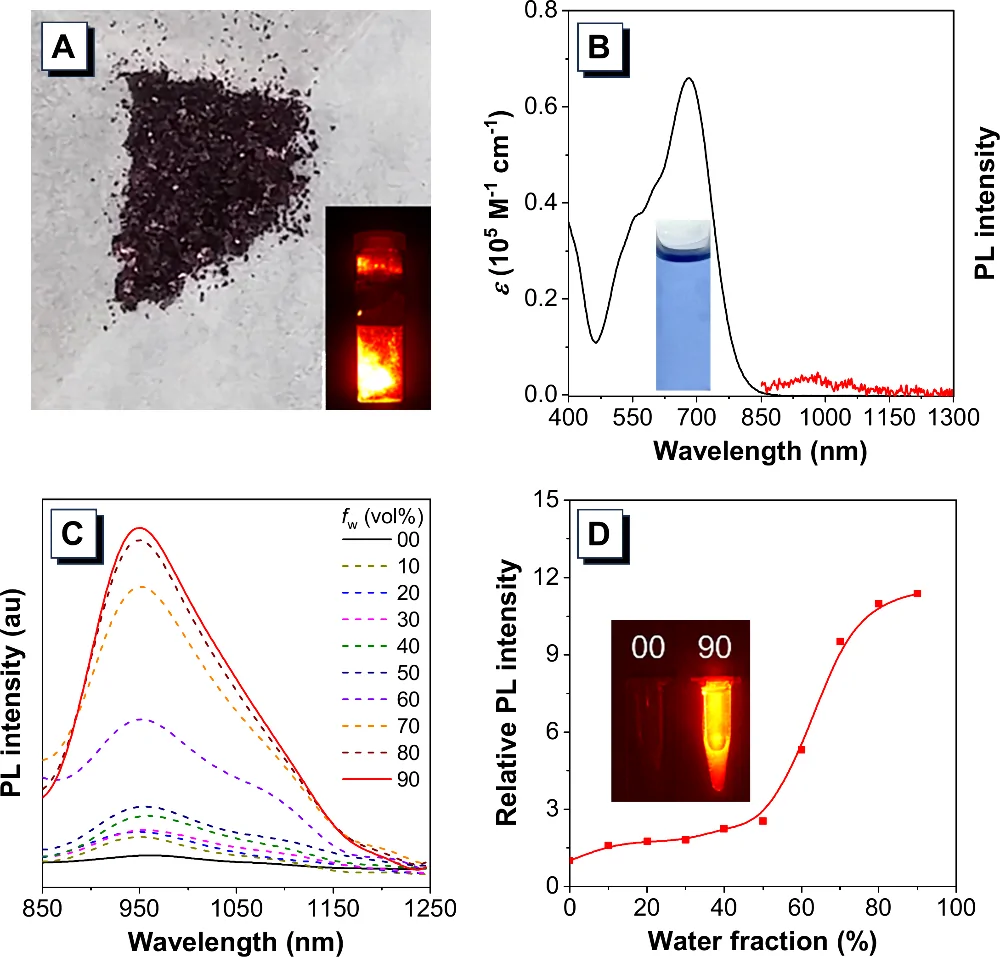
(A) Photograph of TSPCI powders captured under daylight. Inset: Imaging of the TSPCI powders taken under laser illumination of 808 nm. (B) *ε* and fluorescence spectra of TSPCI in THF. Inset: Photograph of TSPCI in THF captured under daylight. (C) Photoluminescence (PL) spectra of TSPCI in THF and THF/water mixtures with the different *f*_w_. (D) Plot of relative PL intensities at maximum emission wavelength versus *f*_w_. Concentration, 10 μM; excitation wavelength, 808 nm. Inset: fluorescence photographs of TSPCI in THF (left) and water/THF mixture (*f*_w_ = 90%) (right) under 808 nm laser illumination, which were collected using an InGaAs camera (1000–1600 nm).

**Figure 2:**
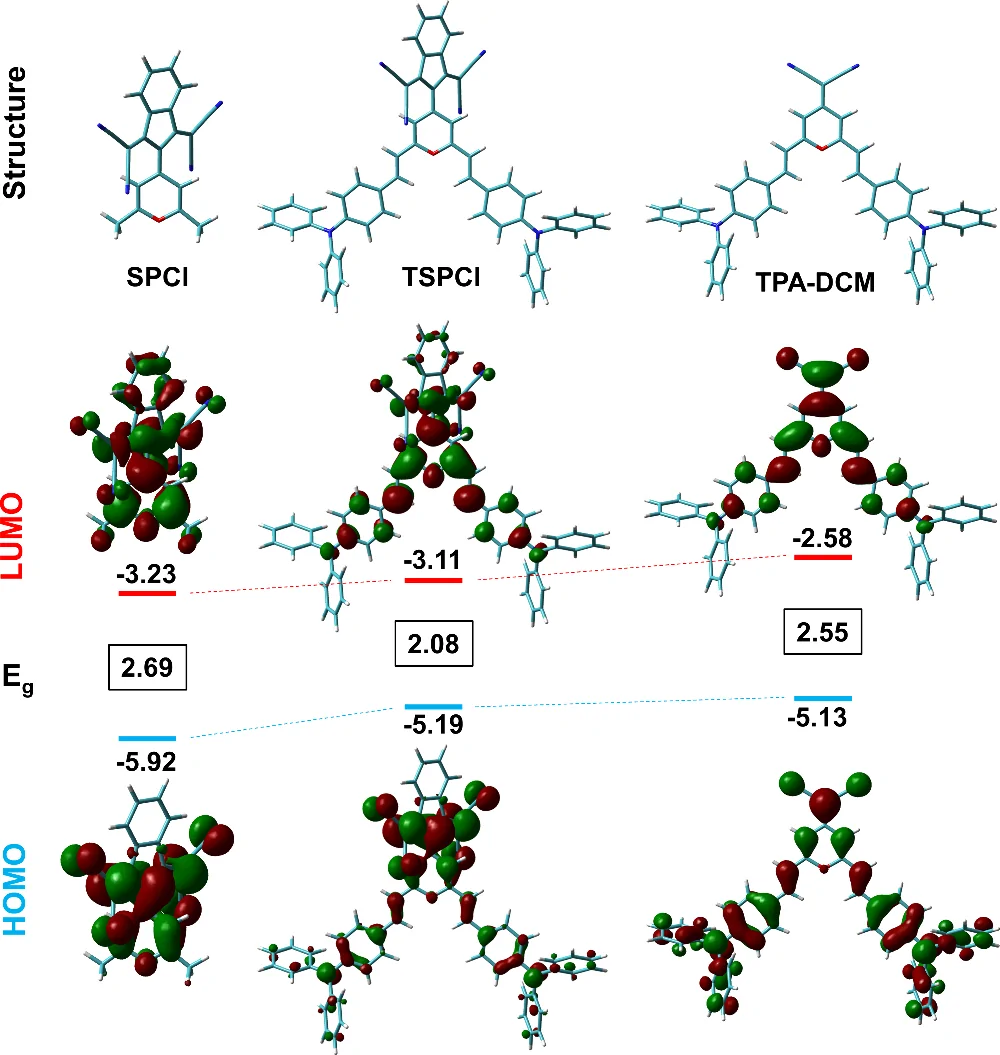
Molecular geometries, orbitals, and energy levels of SPCI, TSPCI, and TPA-DCM in S_0_ calculated with TD-DFT at the level of B3LYP/6-31G* based on solvation of THF, in which vertical excitation as linear response solvation was used.

In contrast to previously reported AIEgens with deep-red or NIR emission, the NIR-II emission of TSPCI in various organic solvents with different polarities was negligible (Figure S12). This interesting phenomenon motivated us to further study the origin of the fluorescent quenching mechanism of TSPCI in the solution state. The theoretical calculations of their optimized molecular geometries and frontier orbitals in the ground (S_0_) and excited (S_1_) states with TD-DFT at the level of B3LYP/6-31G* were calculated by using the polarizable continuum model based on solvation of THF, and their detail data were summarized in Figure 2 and Figures S13–S16 and Tables S2–S6. The electron density in S_0_ of the highest occupied molecular orbital (HOMO) was mainly distributed form the TPA donor to the 2,6-dimethyl-4*H*-pyrane (DMP) spacer, whereas that of the lowest unoccupied molecular orbital (LUMO) was localized from the SPCI to the TPA (Figure 2 and Figure S16). Both of the energy levels of HOMO and LUMO for TSPCI were more up-shifted than that for its precursor SPCI (−5.92 vs −5.19 eV for HOMO and −3.23 vs −3.11 eV for LUMO) owing to the contribution of the electron-donating ability of TPA. Contrarily, TSPCI had more down-shifted energy levels in comparison with TPA-DCM (−5.19 vs −5.13 eV for HOMO and −3.11 vs −2.58 eV for LUMO) after replacing the malononitrile acceptor with a stronger electron-withdrawing ability of BDCI acceptor (Figure 2 and Scheme S1). Thanks to the synergistic D–A contribution, TSPCI had the lowest energy gap (2.08 eV) as well as performed the NIR absorption with high oscillator strength (*f*_OS_, 0.91) in the first vertical transition from S_0_ to S_1_ states, which was in agreement with the experimental data. In contrast, the *f*_OS_ value of the first vertical radiation from S_1_ to S_0_ states was almost zero, implying that the π*−π orbital overlap between HOMO and LUMO in the S_1_ state was ineffective or very low to bring about no emission that could be found in THF solution, which was also consistent with the experimental results (Figure S12).

Based on the molecular geometries of S_0_ and S_1_ states (Figure 3A,B), the dihedral angle and the bond length between the BDCI acceptor and DMP spacer sharply increased from 55.0 to 89.9° and lengthened from 1.463 to 1.475 Å, respectively. Such a double bond is a kind of twisted quasi-double-bond (TQDB) between a typical C–C single-bond and a normal C=C double bond. The TQDB in the excited state separated the electron cloud of HOMO from that of LUMO to cause no or little π*π orbital overlap and an *f*_OS_ value (Figure S16 and Table S3). It seemed to be inconceivable that the bond length of C=C double bond was longer and the torsion angle of it was much larger than a normal one. To further gain insight into the structure–property relationship of TQDB and AIE properties of TSPCI, we tried to get the single crystal of TSPCI, but it failed. Fortunately, we obtained the single crystal of its precursor, SPCI. The single crystal structure of SPCI was carefully analyzed (Figure S17 and Tables S6 and S7). The double bond between BDCI and DMP was delocalized by the electron-withdrawing acceptors, BDCI and pyrane, forming the quasi-double-bond (1.445 Å of bond length), where the dihedral angle was twisted to be 43.3° as a result of the steric hindrance between BDCI and DMP. Such twisting conformation avoids strong intermolecular π–π stacking. The result was consistent with the theoretical calculation (Table S6) and the literature-reported SPCI dimer.^54^ In addition, multiple weak intermolecular interactions were observed, such as CN···H (2.63 Å), CN···C (3.16 Å), CN···O (2.88 Å), NC···H (2.86 Å), and C···C (3.35 Å) (Figure S17). These intermolecular interactions collectively restricted the molecular motions and torsion of TQDB to enhance the π*−π orbital overlap of vertical transition or radiation, bringing about the probability of a radiative rate to promote solid-state emission.

**Figure 3:**
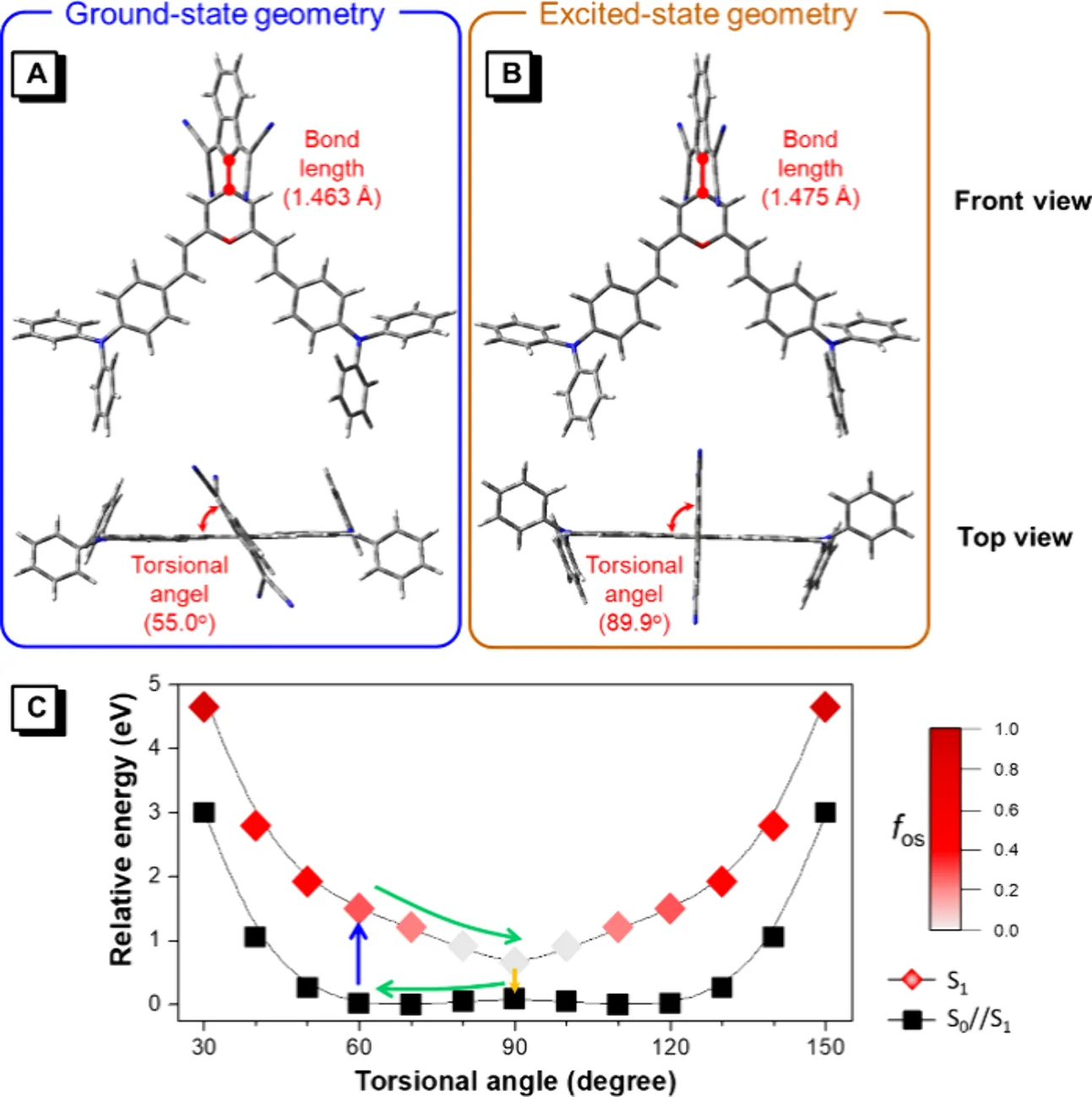
Molecular geometries of TSPCI in (A) ground state and (B) excited state calculated with TD-DFT at the level of B3LYP/6-31G* based on the solvation of THF. Inset: length of the twisted quasi-double-bond (1.463 Å for ground state and 1.475 Å for excited state) and the dihedral angles between the BDCI and DMP (55.0° for ground state and 89.9° for excited state). (C) Potential energy surfaces of rotational double bond for TSPCI, calculated with TD-DFT at the B3LYP/6-31G* level. S_1_ was the energy of the first excited transition, S_0_//S_1_ was the vertical projected energy of the S_0_ state based on the S_1_ geometries, and *f*_OS_ was the oscillator strength of an electronic dipole-allowed transition between S_0_//S_1_ and S_1_ states.

To decipher the AIE mechanism of TSPCI from the solution to aggregated states, the potential energy surface (PES) of their S_1_ states as a function of the dihedral angle between BDCI and DMP was further calculated by scanning the torsional angle from 30 to 150° at 10° intervals (Figure 3C). The *f*_OS_ values were almost zero in the angle range of 80°–100°, in which no or little π*−π orbital overlap causes no vertical radiation from S_1_ to S_0_ states. Thus, the large torsion angle of TSPCI in the S_1_ state consumed the energy of the excited state nonradiatively. In contrast, the torsion angle will be less twisted in the aggregated or solid states and decreased below 80° to be helpful to the transition of π* to π orbitals and the increase of the *f*_OS_ value, leading to the probability of vertical emission and solid luminescence. These calculation results declared the reason why TSPCI shows weak or no emission in solutions but strong fluorescence upon aggregation or in the solid state. Different from the previously published NIR AIEgens in the literature, the rotation of TQDB in the excited state performed better “off–on” AIE features.

Furthermore, we performed some simple experiments to demonstrate the advantages of NIR-II AIEgen over previously reported deep-red/NIR-I AIEgen (TTD, Scheme S1). TTD was chosen to do the comparison because it has a core structure similar to that of TSPCI and has been widely adopted in bio-imaging for several years. Two panels with letters drawn using TTD and TSPCI, respectively, were prepared as depicted in Figure 4A, in which the different numbers of papers was used to cover (the thickness of each paper was 64 μm). Without the paper covered on the panels, sharp and clear images of letters were obtained in both NIR-I and NIR-II spectral windows. Upon increasing the number of papers, the scattering of photons became obvious in the NIR-I region. As a result, the image of letters became smeared and invisible as more than 4 papers covered the panel (Figure 4B and Figure S18), whereas for the NIR-II region imaging, the fluorescent signal of letters retained the distinct and visible signal area, even under the 10 layers of papers with a total thickness of 640 μm (Figure 4C and Figure S19). Fluorescent SBR was quantified by measuring the distance of the full width at half-maximum (fwhm) versus the thickness of the papers covered on the panels (Figure 4D and Figure S20). As the thickness of the paper layers increased from 0 to 576 μm, the fwhm of the NIR-II lettering obtained with TSPCl exhibited only a 0.1-fold increase, indicating minimal broadening and excellent preservation of the imaging resolution. In contrast, under identical conditions, the fwhm of the lettering obtained with the control compound TTD showed a 2.2-fold increase, reflecting significant scattering-induced blurring. Since a smaller fwhm correlates with a higher SBR, the negligible broadening observed for TSPCI demonstrates its superior resistance to photon scattering in turbid media. These results underscore the deep tissue penetration depth and high SBR achievable with TSPCl in the NIR-II window, making it highly advantageous for high-resolution deep-tissue imaging.

**Figure 4:**
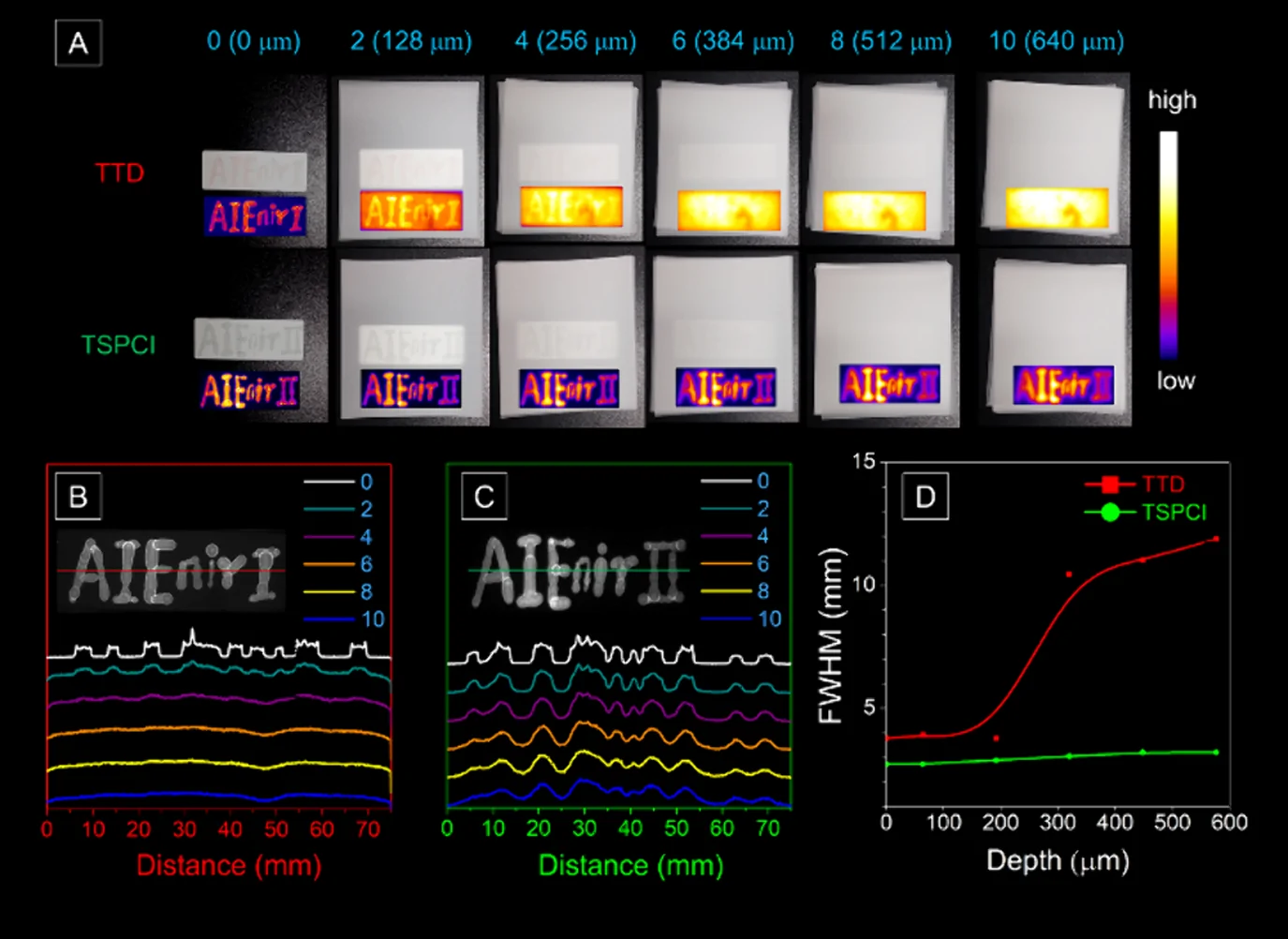
In vitro comparison of the fluorescence imaging performance of NIR-I AIEgen (TTD) and NIR-II AIEgen (TSPCI). (A) Photographs and fluorescent images of two panels with letters drawn using TTD and TSPCI, respectively, covered with different numbers of papers (64 μm per paper) and collected by using a Si-based VIS/NIR-I camera (300–900 nm) and InGaAs-based NIR-II camera (900–1600 nm), respectively. (B) NIR-I fluorescence intensity profiles across the red line of panels, where the “AIEnirI” letters were drawn with TTD and the digital “1–10” indicated the different numbers of covered papers. (C) NIR-II fluorescence intensity profiles across the green line of panels, where the “AIEnirII” letters were drawn with TSPCI. (D) Full width at half-maximum (fwhm) curves of TTD-drawn panel (NIR-I fluorescence) and TSPCI-drawn panel (NIR-II fluorescence) versus the thickness of the papers covered on the panels.

It is also important to evaluate the performance of AIEgens with NIR-II emissions in the application of in vivo imaging. Before evaluating its in vivo imaging performance, the optical properties and stability of TSPCI under physiological conditions were first examined. As shown in Figure S21, TSPCl exhibits an absorption maximum at 663 nm and an emission maximum at 950 nm in a PBS solution, corresponding to a large Stokes shift of 287 nm. This large Stokes shift is particularly advantageous for in vivo imaging applications, as it minimizes self-absorption, reduces interference from excitation light, and improves SBR. Upon continuous irradiation with an 808 nm laser, TSPCI demonstrated excellent photostability, with negligible degradation in absorbance intensity over time in both water and 10% fetal bovine serum (FBS) solution (Figure S22). Furthermore, its fluorescence intensity was enhanced under weakly acidic conditions and remained consistently bright in the presence of various interfering ions (Figure S23). Notably, an increase in FBS content further promoted NIR-II emission compared with that in PBS alone (Figure S24). The MTT assay and liver and kidney functions were further employed to evaluate the biological safety of TSPCI. The MTT assay on NIH-3T3 cells demonstrated that the cell viability remained above 90% even at concentrations up to 100 μM (Figure S25). All liver and kidney function indicators remained consistent after the injection of TSPCI compared with the injection of PBS, suggesting good biological safety (Table S8). These results collectively indicate that TSPCI possesses stable and robust NIR-II imaging capability under complex physiological conditions. In order to better understand the advantages of NIR-II imaging, TTD and TSPCI solutions were injected into each side of a nude mouse, respectively (Figure S26). The whole-body imaging of nude mice was acquired by using Si-based NIR-I and InGaAs-based NIR-II cameras, respectively. We found that the signal intensity of TTD was only higher ∼1.5-fold compared to that in the skin tissue around the injection site. In contrast, the signal-to-background ratio (S/N ratio) was as high as ∼11-fold for TSPCI (Figure S26D). The enhanced S/N ratio for NIR-II imaging should be mainly attributed to the significant decrease of autofluorescence signals of the mouse body in the NIR-II relative to NIR-I (Figure S26E).

We further carried out NIR-II imaging comparative experiments to demonstrate the advantage of TSPCI in comparison with IR-1048 (a traditional commercial NIR-II polymethine dyes). As shown in Figure 5A–D, under 808 nm laser illumination (with a power density of 6.4 mW/cm^2^, which is significantly lower than the safe-exposure limit of 330 mW/cm^2^ at 808 nm for small animal experiments), IR-1048 powder was non-emissive as a fact of ACQ effect, while TSPCI powder showed a bright NIR-II fluorescence. In the experiment of monitoring molecular aggregation (Figure S27), IR-1048 showed strong luminescence in a dilute solution of DMSO; however, its emission became weakened and even totally quenched upon the addition of water. By contrast, TSPCI showed no emission in dilute THF solution but strong fluorescence in the aqueous medium due to the molecular aggregation restricted the torsion of TQDB of TSPCI molecules to increase the probability of emission rate radiatively. The unique responsive capacity to external environmental variations of TSPCI inspired us to evaluate the potential of NIR-II AIEgens as microenvironment-sensitive activate probes for in vivo dynamic imaging and monitoring. The hydrophobic interaction drives nonpolar solutes to aggregate in aqueous environments and plays a critical role in many fundamental bioprocesses. As a proof of concept, we tried to monitor the hydrophobic aggregation in vivo by using as-prepared NIR-II AIEgen TSPCI. TSPCI solution (non-emissive in the dissolved state) was intraperitoneally injected into the mouse to visualize the aggregation process in vivo, where molecular aggregation is an intrinsic process for hydrophobic species in aqueous media. The hydrophobic interactions drove TSPCI to aggregate in aqueous medium, and the fluorescence was enhanced along with the prolonged time (Figure 5E,F). On the other hand, the bright NIR-II fluorescence of IR-1048 disappeared after intraperitoneal injection (Figure 5G,H). In order to enable a side-by-side comparison between TSPCI and IR-1048, their solutions were injected simultaneously into each side of a nude mouse. The results showed that one aspect waned and the other waxed (Figure 5I–L). Next, we performed whole-body imaging of nude mice after intravenous injection of TSPCI in PBS buffer (Figure S28). Once TSPCI aggregates were injected, fluorescence images over the NIR-II spectrum region were collected continuously to track the blood circulation of TSPCI in real time. The pulmonary and systemic circulation could be clearly distinguished from the video-rate imaging, indicating that TSPCI could be used as a promising NIR-II contrast agent for in vivo dynamic imaging. To further explore the potential applications of TSPCI, its capability for lymph node mapping was evaluated. TSPCI was administered into the left and right footpads of mice 30 and 5 min prior to imaging, respectively (Figure S29). While lymph nodes on both sides were visualized, those on the left side exhibited markedly stronger fluorescence as a result of the longer postinjection accumulation time. These results demonstrate the excellent lymph node mapping ability of TSPCI.

**Figure 5:**
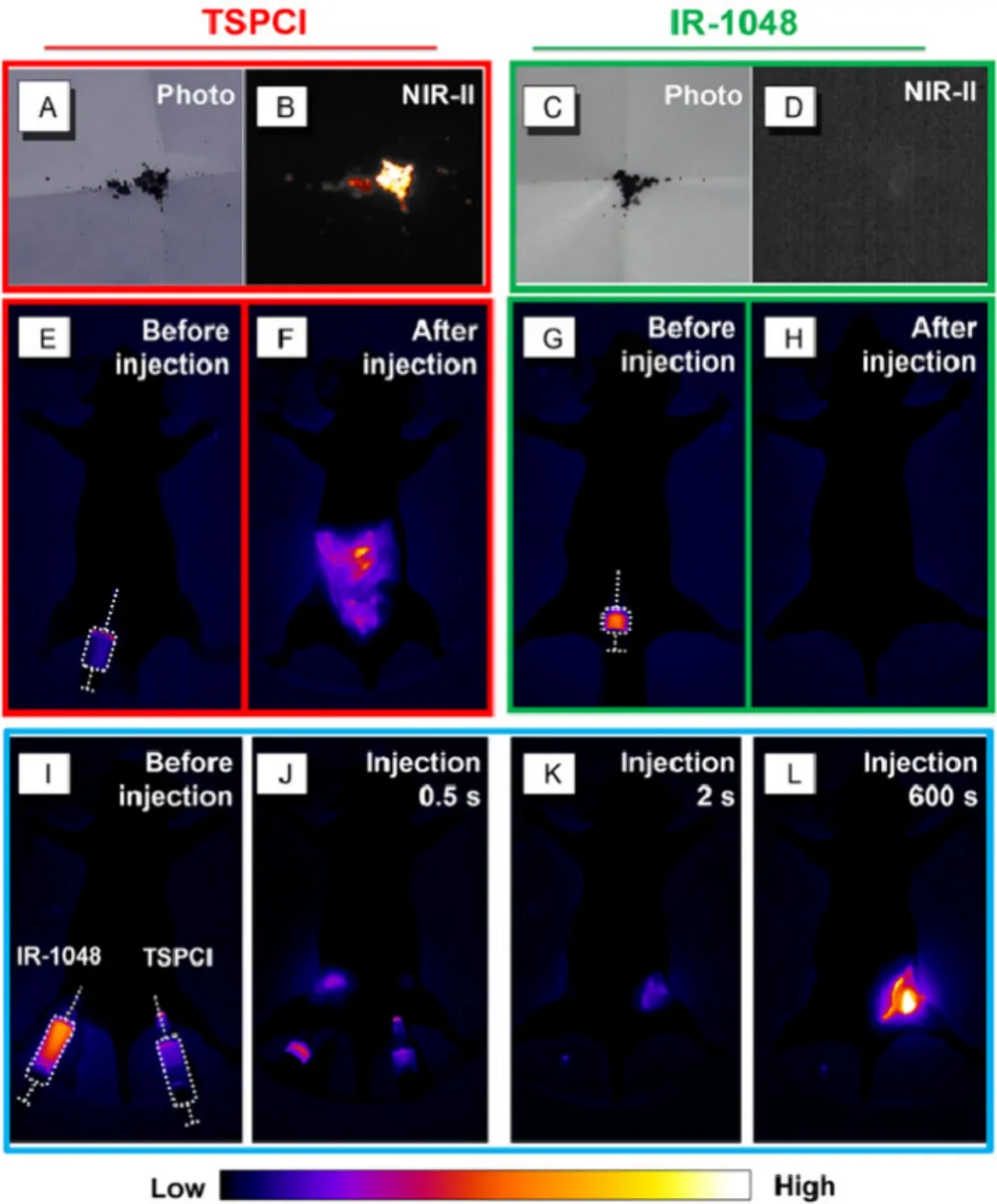
Comparison of NIR-II fluorescence properties of TSPCI (AIEgen) and IR-1048 (ACQ fluorophore). (A) Photograph and (B) NIR-II imaging of TSPCI powders. (C) Photograph and (D) NIR-II imaging of IR-1048 powders. (E and F) NIR-II imaging of the nude mouse before and after intraperitoneal injection of TSPCI solution. (G and H) NIR-II imaging of the nude mouse before and after intraperitoneal injection of IR-1048 solution. (I–L) Time-dependent NIR-II fluorescence images of the nude mouse before and after intraperitoneal injection of IR-1048 (left side) and TSPCI (right side) solution simultaneously.

In summary, we have designed and synthesized a novel “simple” structural NIR-II fluorophore (TSPCI) with typical AIE features by a facile two-step procedure. As-prepared AIEgen showed no emission in solution, but strong NIR-II fluorescence in aggregated and solid states stemmed from the TQDB property in accordance with the results of theoretical calculations, where TQDB between donor and acceptor played the crucial role in the fluorescent quenching of TSPCI in the solution state. This result suggested that the TQDB property not only paves a convenient avenue to design NIR-II materials with AIE attributes but also performs in a “turn-on” manner. Preliminary studies in imaging reveal the great potential of TSPCI as a building block with superior responsive ability to easily fabricate NIR-II activatable fluorescent probes. In the future, we will focus on the development of novel “smart” NIR-II molecule probes based on TSPCI scaffold for in vivo dynamic functional bio-imaging.

## Supplementary Material



## Notes

Materials and methods, synthetic procedures, characterization and computational details, including Figures S1–S29 and Tables S1–S8 (PDF)

## Abbreviations

AIE: aggregation-induced emission
AIEgen: luminogen with AIE attribute
