# Supplementary material for: Donor–Acceptor Bridging via Twisted Quasi-Double-Bond: A Facile Design Strategy for Typical NIR-II AIEgen with “Simple” Structure
Source: Chem Biomed Imaging. 2026 Apr 8;4(9):1987–95. doi: 10.1021/cbmi.5c00302 (PMC13625704; doi:10.1021/cbmi.5c00302)
Supplement: Supplementary file 1 [file im5c00302_si_001.pdf]

Supporting Information

Donor-Acceptor Bridging via Twisted Quasi-Double-Bond: A Facile Design Strategy for Typical NIR-II AIEgen with "Simple" Structure

Chunbin Li,[a,f‡] Jen-Shyang Ni,[d,‡] Pengfei Zhang,[a,c,‡,*] Daoyong Jiang,[e] Jianguo Wang,[f] Kai Li,[g] Shuang-Quan Zang,[g] Ryan T. K. Kwok,[a] Weihong Tan,[h] Ben Zhong Tang[a,b,*]

[a] Department of Chemistry, The Hong Kong Branch of Chinese National Engineering Research Center for Tissue Restoration and Reconstruction, State Key Laboratory of Nervous System Disorder, Division of Life Science, and Department of Chemical and Biological Engineering, The Hong Kong University of Science and Technology, Kowloon, Hong Kong 999077, China

[b] Guangdong Basic Research Center of Excellence for Aggregate Science, School of Science and Engineering, Shenzhen Institute of Aggregate Science and Technology, The Chinese University of Hong Kong, Shenzhen (CUHK-Shenzhen), Shenzhen 518172, China. E-mail: tangbenz@cuhk.edu.cn

[c] Guangdong Key Laboratory of Nanomedicine, CAS Key Laboratory of Health Informatics, Shenzhen Bioactive Materials Engineering Lab for Medicine, Institute of Biomedicine and Biotechnology, Shenzhen Institutes of Advanced Technology, Chinese Academy of Sciences, Shenzhen 518055, China. E-mail: pf.zhang@siat.ac.cn

[d] Department of Biomedical Engineering, Southern University of Science and Technology, Shenzhen, Guangdong, 518055 China

[e] School of Chemistry and Life Sciences, Guilin Normal University, Guilin, 541199, China

[f] College of Chemistry and Chemical Engineering, College of Biomedical Sciences, Inner Mongolia Key Laboratory of Synthesis and Application of Organic Functional Molecules, Inner Mongolia University, Hohhot 010021, China

[g] College of Chemistry and Molecular Engineering, Zhengzhou University, Science Road No. 100, Zhengzhou 450001, China

[h] Hangzhou Institute of Medicine (HIM), Chinese Academy of Sciences, Hangzhou, Zhejiang 310022, China.

‡ These authors contributed equally to this work.

Experiment section:

Materials and characterization.

Unless otherwise noted, all reagents were purchased from commercial suppliers and used without further purification. All solvents were of analytical grade or purified using standard methods prior to use. Ultrapure water (18.25 MΩ cm, 25 °C) was used to prepare all aqueous solutions for in vitro experiments. PBS buffer (pH 7.4) was used to prepare all aqueous solutions in vivo experiments. The TTD and TPA-DCM compounds were kindly provided by AIEgen Biotech Co., Ltd. The ^{1}H and ^{13}C NMR spectra were obtained on Bruker AV-400 or Bruker AV-600 spectrometer by using tetramethylsilane (TMS) as an internal standard and $CDCl_3$, THF-d_8, 1,2-Dichloroethane-d_4 or DMSO-d_6 as the solvent. High resolution mass spectra (HRMS) were obtained using a GCT premier CAB048 mass spectrometer operating in MALDI-TOF mode. The absorption spectra were measured on a PerkinElmer Lambda 25 UV-Vis absorption spectrophotometer. Photoluminescence (PL) spectra were recorded with Edinburgh F900 fluorescent spectrometer equipped with xenon arc lamp. Single-crystal X-ray diffraction data were collected on a Bruker SMART APEX CCD diffractometer using graphite-monochromatized Mo Kα radiation (λ = 0.71073 Å) at room temperature using the ω-scan technique. Simulation was carried out with the Gaussian 09 package.

Synthesis and characterization of SPCI

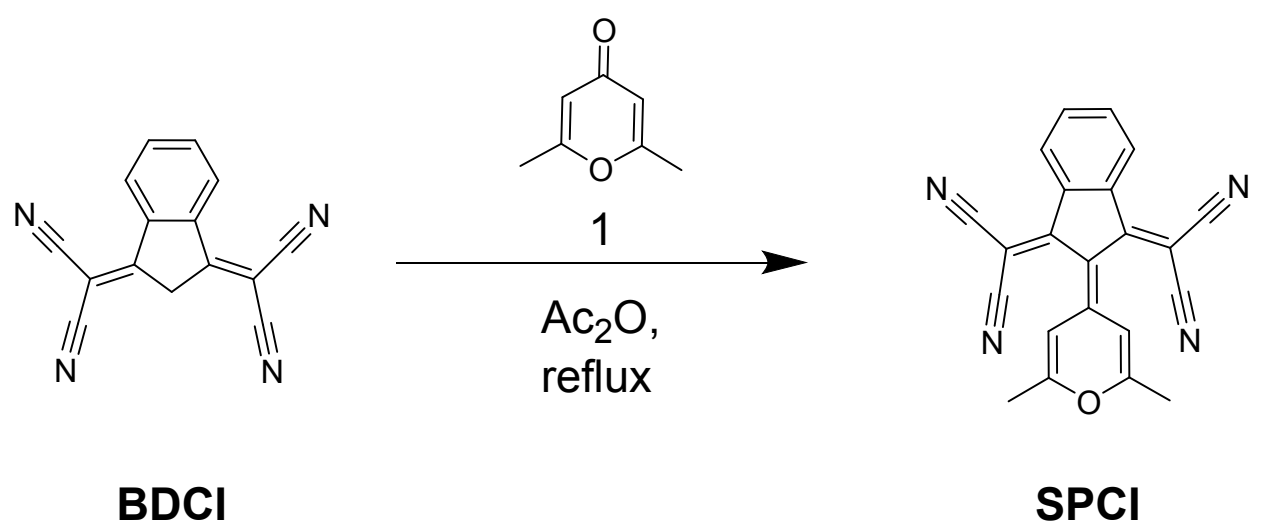

In a 50 mL two-necked flask, the mixture of 1 (2,6-disubstituted y-pyrones, 300 mg, 2.41 mmole), BDCI (1,3-bis(dicyanomethylene) indan, 648 mg, 2.63 mmole), and acetic anhydride (10 ml, 97.8 mmole) was refluxed overnight. Then, the solution was cooled to room temperature and filtered to obtain the crude product, which was purified by silica column chromatography (eluent solvent, dichloromethane: hexane = 1: 1, *V*/*V*) to obtain compound SPCI as a purple powder (626 mg), yield 74%. ^{1}H NMR (400 MHz, DMSO-d_6) δ: 8.34 (br, 2H), 7.87 (s, 2H), 7.68 (br, 2H), 2.81 (s, 6H). ^{13}C NMR (100 MHz, $CDCl_3$) δ: 169.03, 158.11, 154.08, 137.53, 131.73, 123.57, 120.11, 115.56, 115.53, 110.58, 61.14, 20.49. HRMS (MALDI-TOF), *m*/*z*: $[M]^+$ calculated: 348.1011, found: 348.1037.

Synthesis and characterization of TSPCI

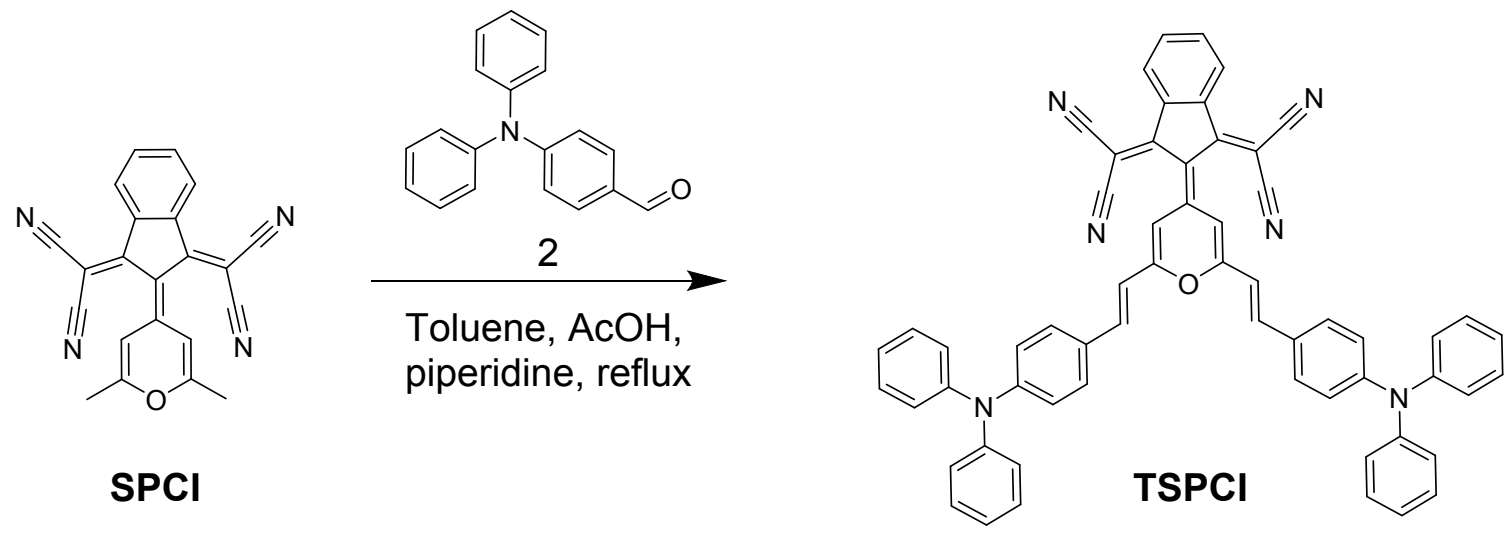

In a 50 mL two-necked flask, SPCI (168 mg, 0.48 mmol) and 4-(N,N-diphenylamino) benzaldehyde (compound 2, 329 mg, 1.21 mmol) were dissolved in anhydrous toluene (15 mL), then piperidine (1.0 mL) and acetic acid (0.5 mL) were added to the aforementioned mixture[1]. The solution was stirred and heated at 120°C for 8 h, while the solution color changed from purple to dark blue. Then, the solution was cooled to room temperature and filtered to obtain the crude product, which was purified by silica column chromatography (eluent solvent, dichloromethane: methanol = 100: 1, *V*/*V*) to obtain TSPCI as a black powder (47 mg), yield 4%. ^{1}H NMR (400 MHz, THF-d_8) δ: 8.44 (dd, *J*=3.2 Hz, *J*=6.0 Hz, 2H), 8.02 (d, *J*=16.0 Hz, 2H), 7.63 (d, *J*=8.8 Hz, 8H), 7.51 (dd, *J*=3.2 Hz, *J*=5.6 Hz, 2H), 7.35–7.31 (m, 8H), 7.22–7.12 (m, 14H), 6.99 (d, *J*=8.4 Hz, 2H). ^{13}C NMR (100 MHz, $CDCl_3$) δ: 163.72, 156.25, 153.49, 150.87, 145.48, 142.14, 137.50, 130.93, 129.88, 129.10, 126.04, 125.50, 124.47, 123.14, 119.79, 116.78, 116.21, 113.83, 110.69, 57.29. HRMS (MALDI-TOF), *m*/*z*: $[M]^+$ calculated: 858.3107, found: 858.3108.

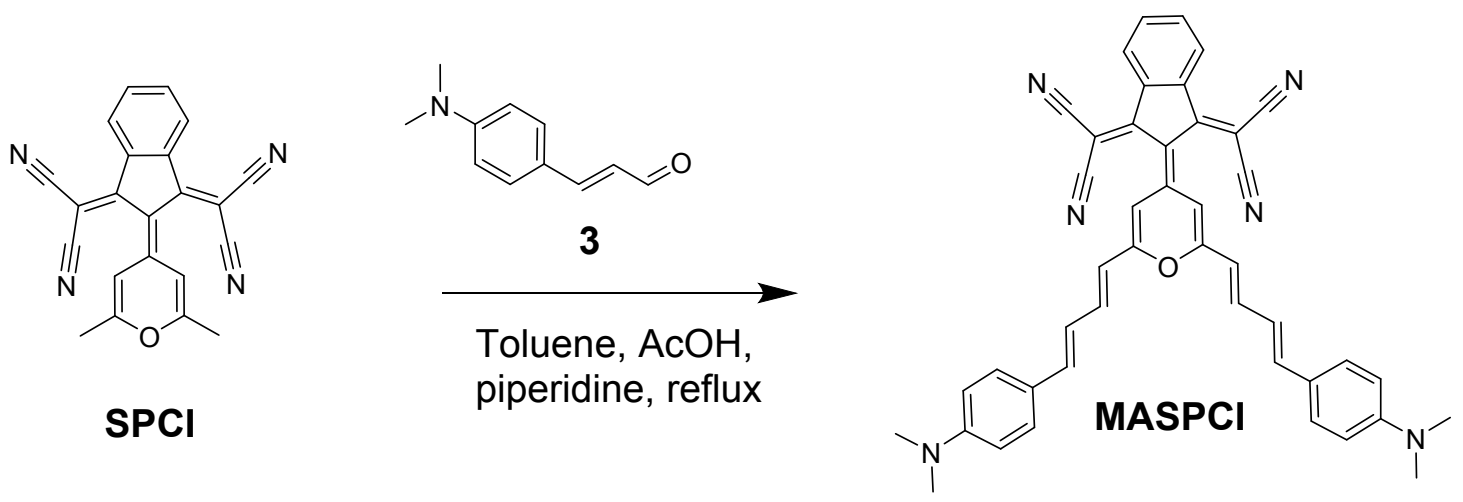

In a 50 mL two-necked flask, SPCI (175 mg, 0.50 mmol) and 4-dimethylaminocinnamaldehyde (compound 3, 263 mg, 1.50 mmol) were dissolved in anhydrous toluene (15 mL), then piperidine (1.0 mL) and acetic acid (0.5 mL) were added to the aforementioned mixture. The solution was stirred and heated at 120 °C for 8 h, while the solution color changed from purple to dark blue. Then, the solution was cooled to room temperature and filtered to obtain the crude product, which was purified by silica column chromatography (eluent solvent, dichloromethane: methanol = 100: 1, V/V) to obtain MASPCl as black powder (99 mg), yield 15%. ^{1}H NMR (600 MHz, 1,2-Dichloroethane-d_4) δ 8.45 – 8.38 (m, 2H), 7.71 – 7.61 (m, 2H), 7.49 (d, *J* = 7.9 Hz, 6H), 7.17 (d, *J* = 15.0 Hz, 2H), 6.98 – 6.86 (m, 4H), 6.72 (d, *J* = 8.5 Hz, 4H), 6.47 (d, *J* = 14.8 Hz, 2H), 3.08 (s, 12H).

Determination of NIR-II Photoluminescence quantum yield of TSPCI.

The NIR-II photoluminescence quantum yield (QY) of TSPCI in aggregate state was measured using commercial fluorescent IR-26 dye as the NIR-II reference standard sample ($QY_{IR\text{-}26}$ = 0.5 % in 1, 2-dichloroethane) according to a previous reported method. The IR-26 was dissolved in 1, 2-dichloroethane (DCE), and diluted to different concentration. The fluorescence spectra in the range of 900 to 1600 nm was collected under the 820 nm excitation. The absorption and emission of TSPCI in the mixture of THF/water (10/90) were measured using the same method with IR-26. Then all emission spectra of both IR-26 and the TSPCI were integrated from 900 to 1600 nm. The integrated NIR-II fluorescence intensity was plotted against absorbance at the excitation wavelength of 820 nm and fitted into a linear function. The slope of TSPCI was compared with NIR-II dye IR-26, and the quantum yield of TSPCI was determined by following equation,

$$QY_{TSPCI} = QY_{IR-26} \frac{I_{TSPCI}}{I_{IR-26}} \frac{A_{IR-26}}{A_{TSPCI}} \frac{n_{TSPCI}^2}{n_{IR-26}^2}$$

Where $n_{\text{TSPCI, IR-26}}$ is the refractive index, $A_{\text{TSPCI, IR-26}}$ is the absorption, and $l_{\text{TSPCI, IR-26}}$ is the integrated

fluorescence signal for the TSPCI and IR-26, respectively.
The quantum yield of TSPCI was determined to be 0.7 %, using IR-26 as a reference (with a nominal QY = 0.5%)

MTT assay:

NIH-3T3 cells were seeded in a 96-well plate at a density of 5000 cells/well and incubated overnight for adhesion. The medium was then replaced with fresh medium containing various concentrations of TSPCl (0–100 μM) and incubated for 20 h. After treatment, 20 μL of MTT solution (5 mg/mL in PBS) was added to each well and incubated for an additional 4 h. The supernatant was carefully removed, and the resulting formazan crystals were dissolved in 150 μL of DMSO. Absorbance was measured at 490 nm using a microplate reader. Cell viability was calculated as the percentage ratio of the absorbance of treated cells to that of untreated control cells.

Animal experiments:

Female BALB/c nude mice (weighed 20-22 g and 5-6 weeks old) were purchased from Vital River Laboratory Animal Technology Co. Ltd (Beijing, China) and all animals received care in compliance with the guidelines outlined in the guide for the Care and Use of Laboratory Animals. The procedures were approved by Animal Care and Use Committee of Shenzhen Institutes of Advanced Technology, Chinese Academy of Sciences (Approval No. YSB-20250317-ZPF-A3575).

In vivo and ex vivo Imaging study:

TSPCI and IR-1048 were prepared as powders and dissolved in appropriate solvents (DMSO for IR-1048, THF for TSPCI) at a concentration of 1 mM. All NIR-II imaging experiments were performed using an InGaAs camera-equipped NIR-II imaging system under 808 nm laser excitation at a power density of 6.4 mW/cm² (significantly lower than the safety limit of 330 mW/cm² for small animal experiments at 808 nm). NIR-I fluorescence imaging was performed using the Maestro in vivo imaging system (CRI, Inc.). To acquire fluorescence of TTD, A 605 nm (±25 nm) bandpass filter and a 645 nm longpass filter were selected as the excitation filter and the emission filter, respectively. NIR-II imaging was performed using the home-built NIR-II in vivo imaging system. The excitation light was provided by an 808 nm diode laser. The emitted light from the TSPCI was filtered through a 1000 nm long-pass filter, which was coupled with a two-dimensional InGaAs array camera (with spectral response range of 900-1700 nm, Photonic Science) for NIR-II fluorescence signal collection. The excitation power density at the imaging plane was 6.4 mW/cm^2, much lower than the safe exposure limit of 330 mW/cm^2 at 808 nm determined by the International Commission on Nonionizing Radiation Protection. The exposure time for all images was 100 ms.

Lymph node imaging procedure:

For lymph node imaging, mice were anesthetized with isoflurane and positioned on a heating pad to maintain body temperature. TSPCI solution (20 μL, 100 μM in PBS containing 10% DMSO) was subcutaneously injected into the left and right footpads at different time points prior to imaging (e.g., 30 min for the left paw and 5 min for the right paw). NIR-II fluorescence imaging was performed using an InGaAs camera equipped with a 1000 nm long-pass filter. Images were acquired under 808 nm laser irradiation.

Supplementary Figures:

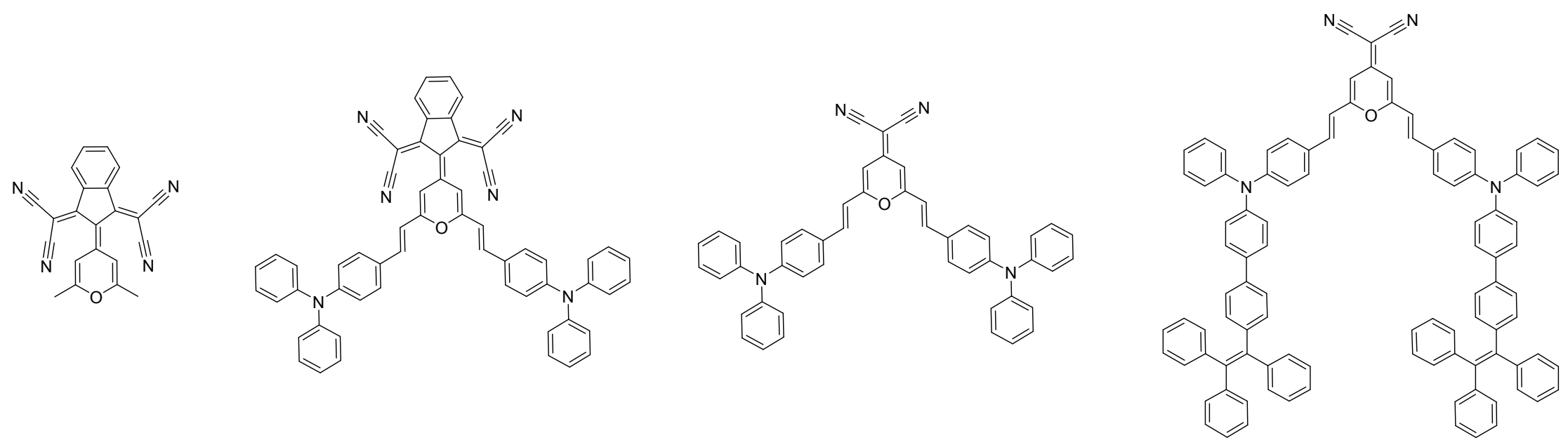

SPCI TSPCI TPA-DCM TTD

Scheme S1. The chemical structures of SPCI, TSPCI, TPA-DCM and TTD.

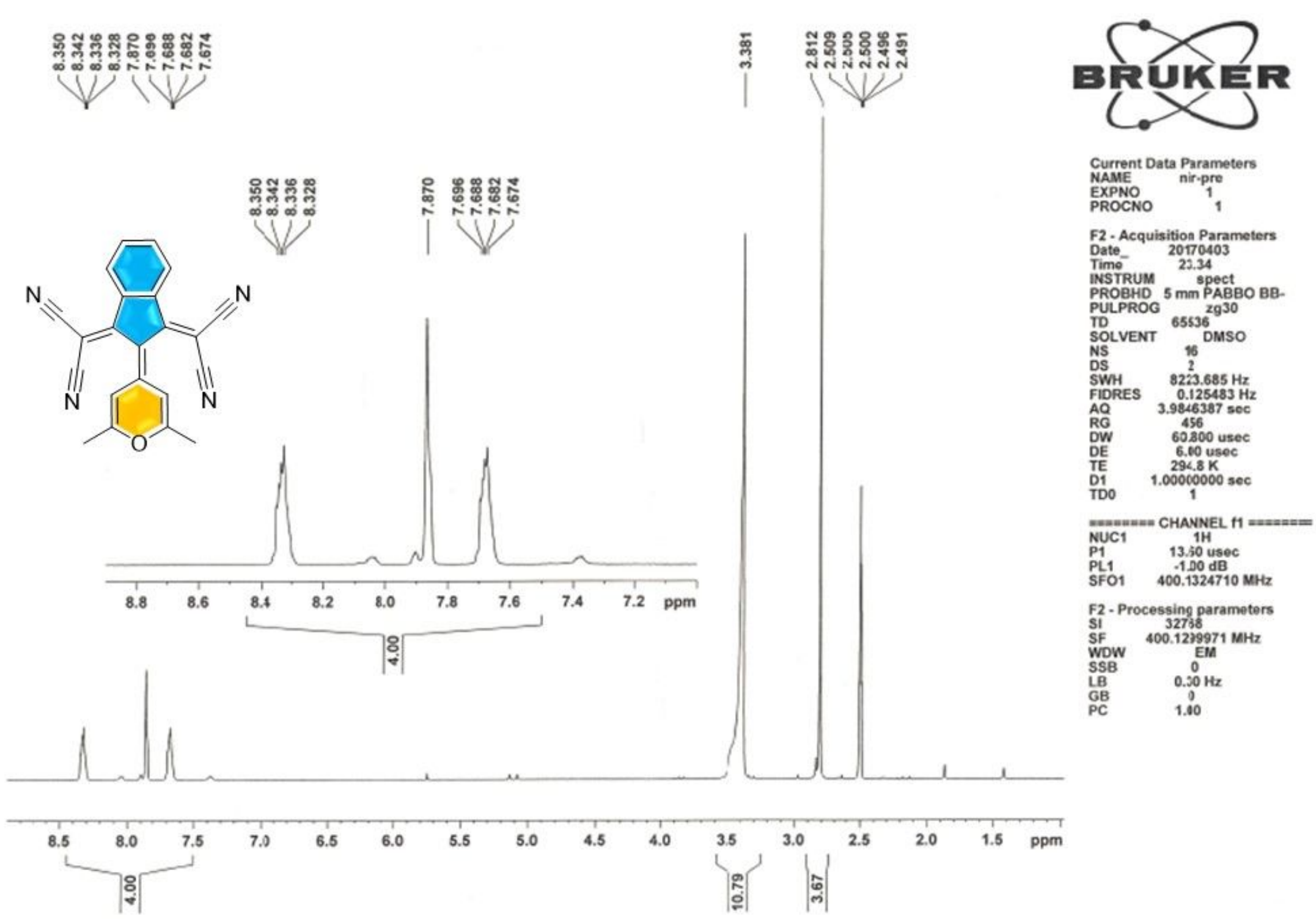

Figure S1. ^{1}H NMR spectrum of SPCI in DMSO-d_6.

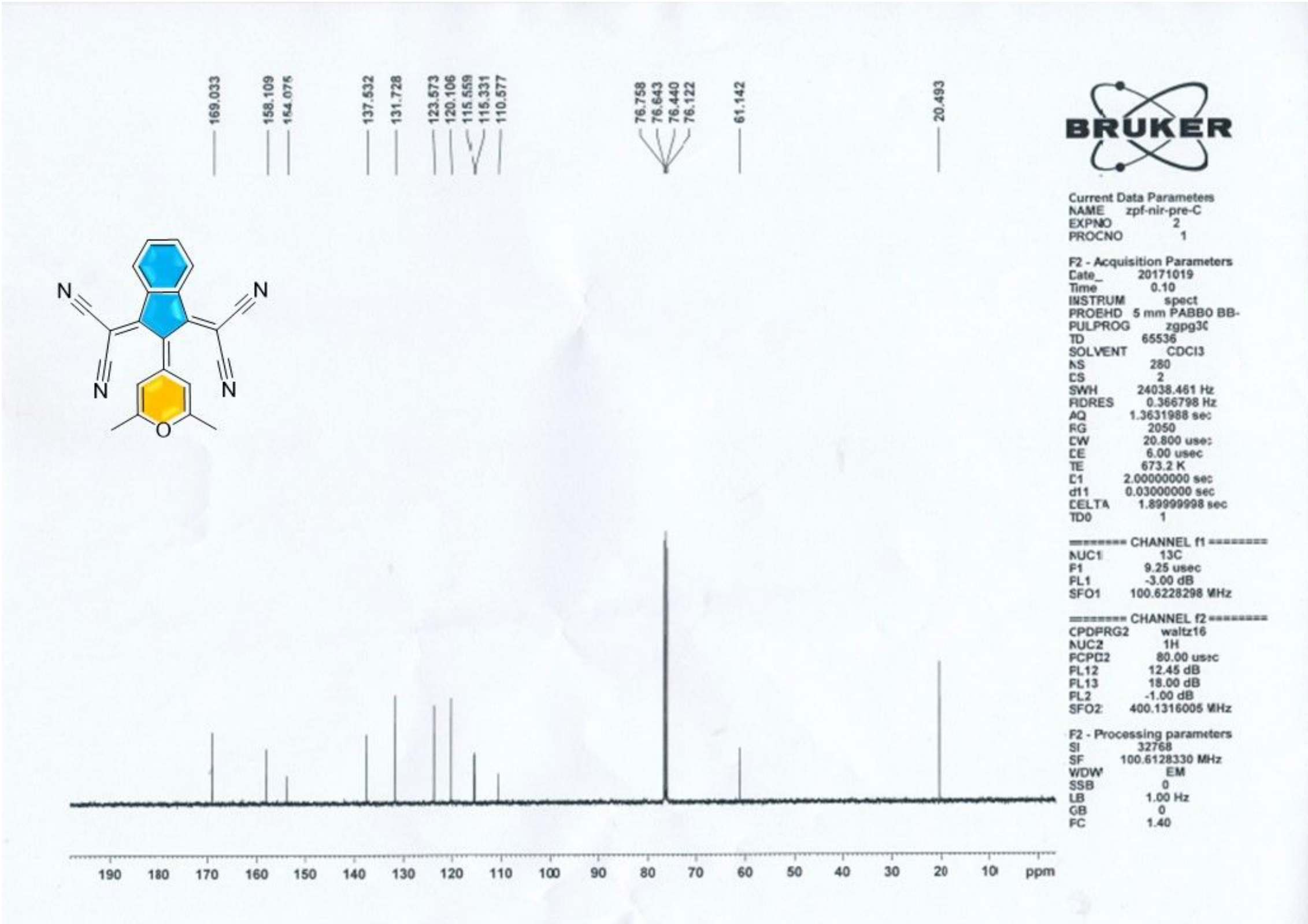

Figure S2. ^{13}C NMR spectrum of SPCI in $CDCl_3$.

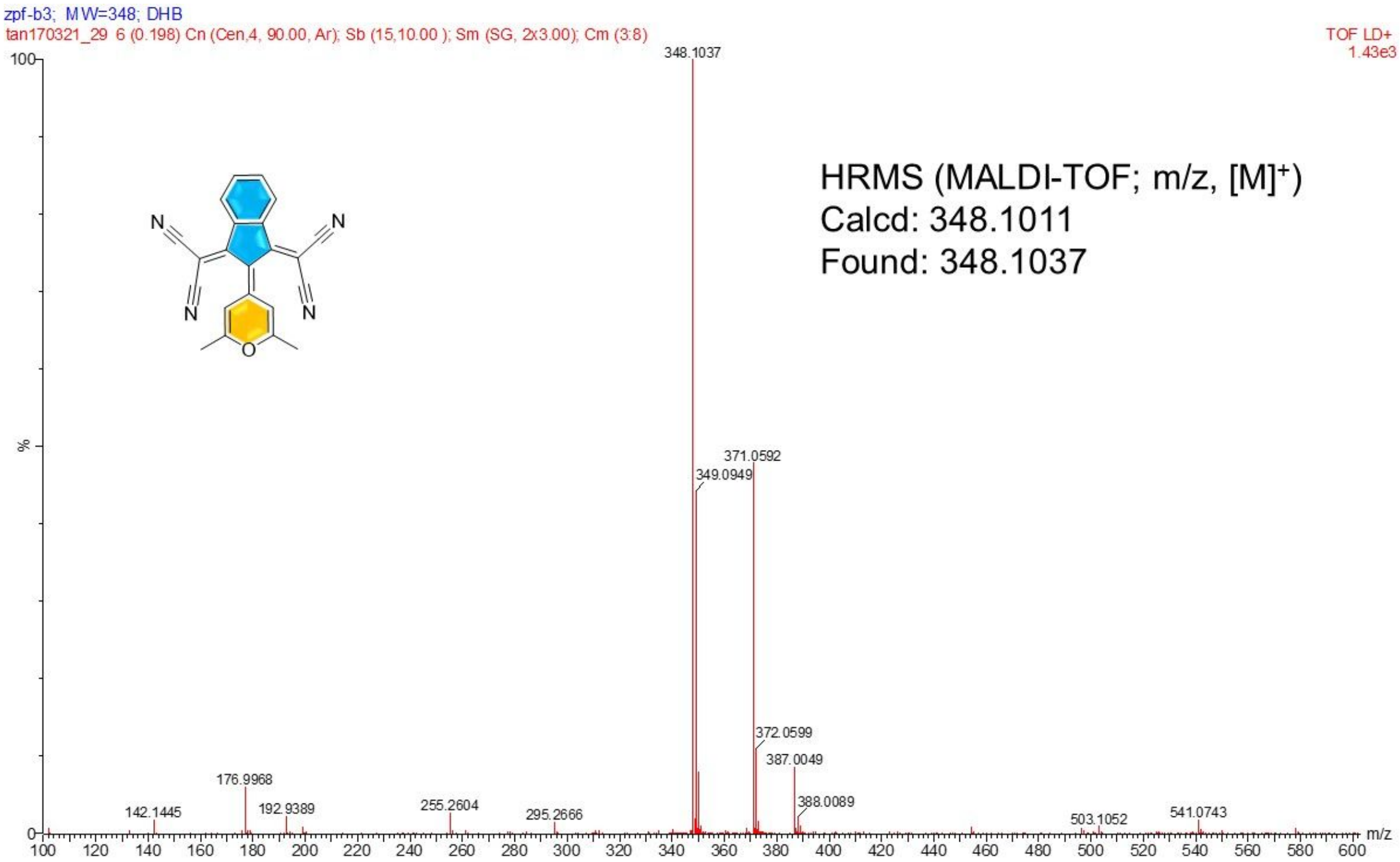

Figure S3. High resolution mass spectrum (MALDI-TOF) of SPCI.

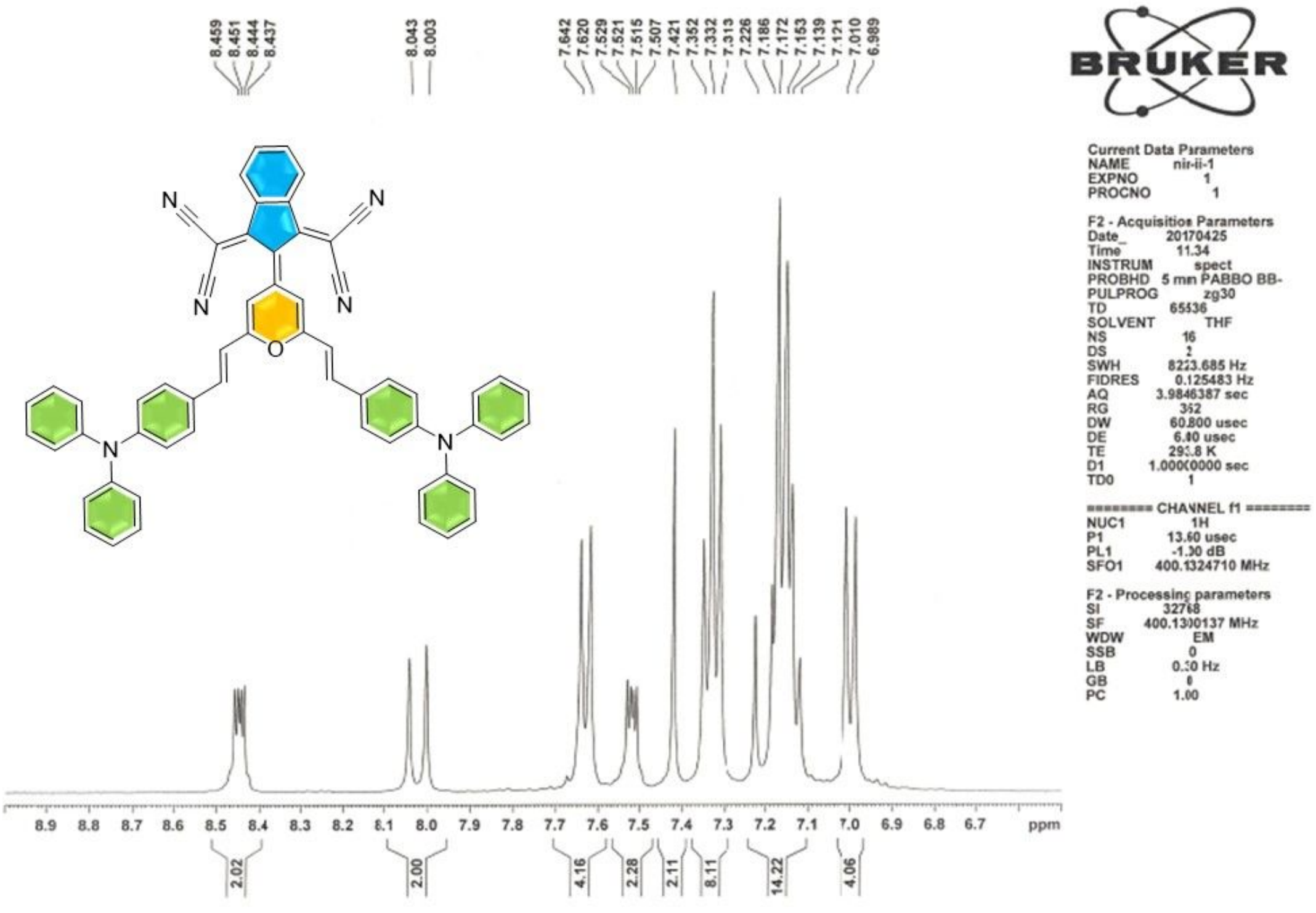

Figure S4. ^{1}H NMR spectrum of TSPCI in THF-d_8.

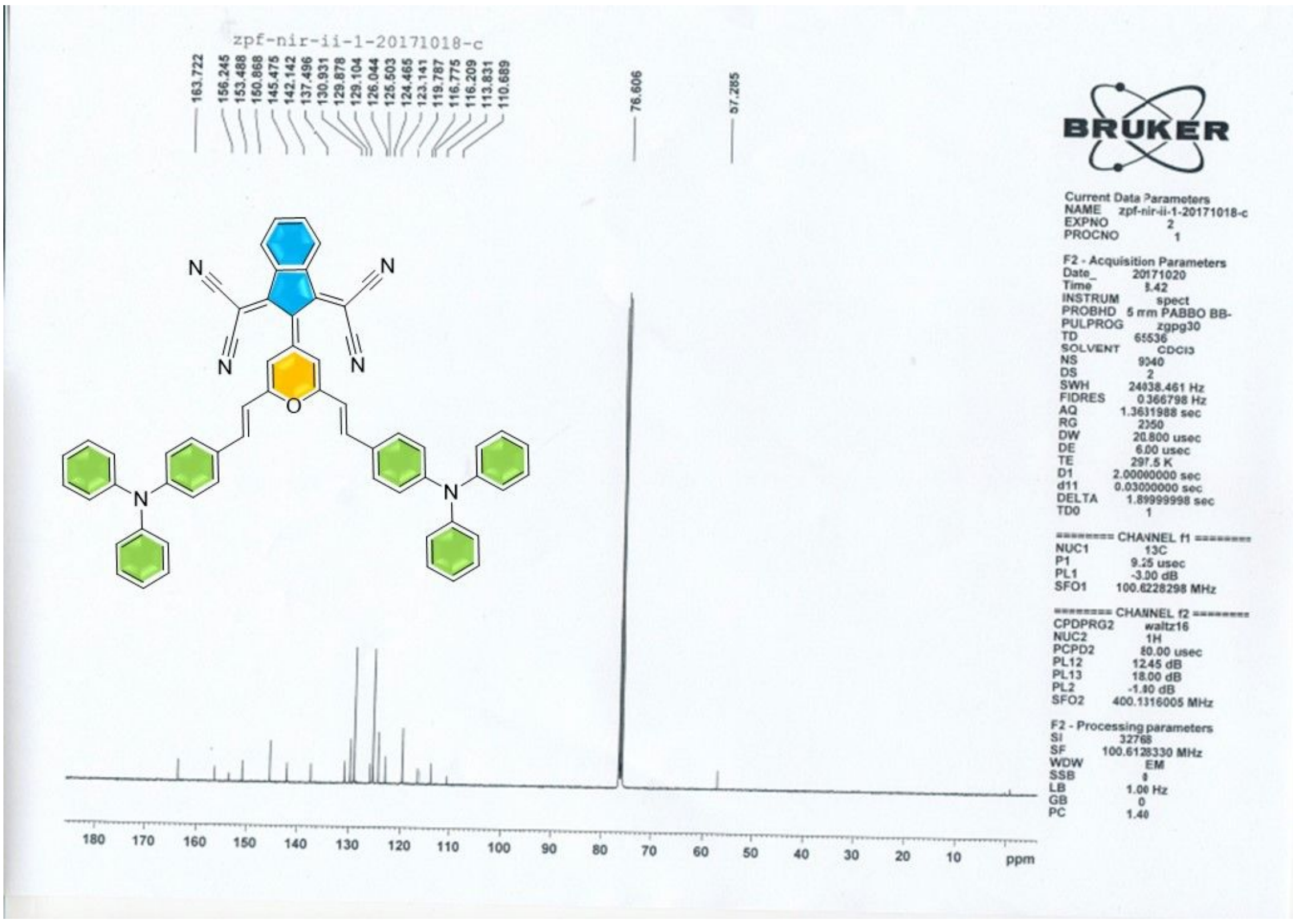

Figure S5. ^{13}C NMR spectrum of TSPCI in CDCl$_3$.

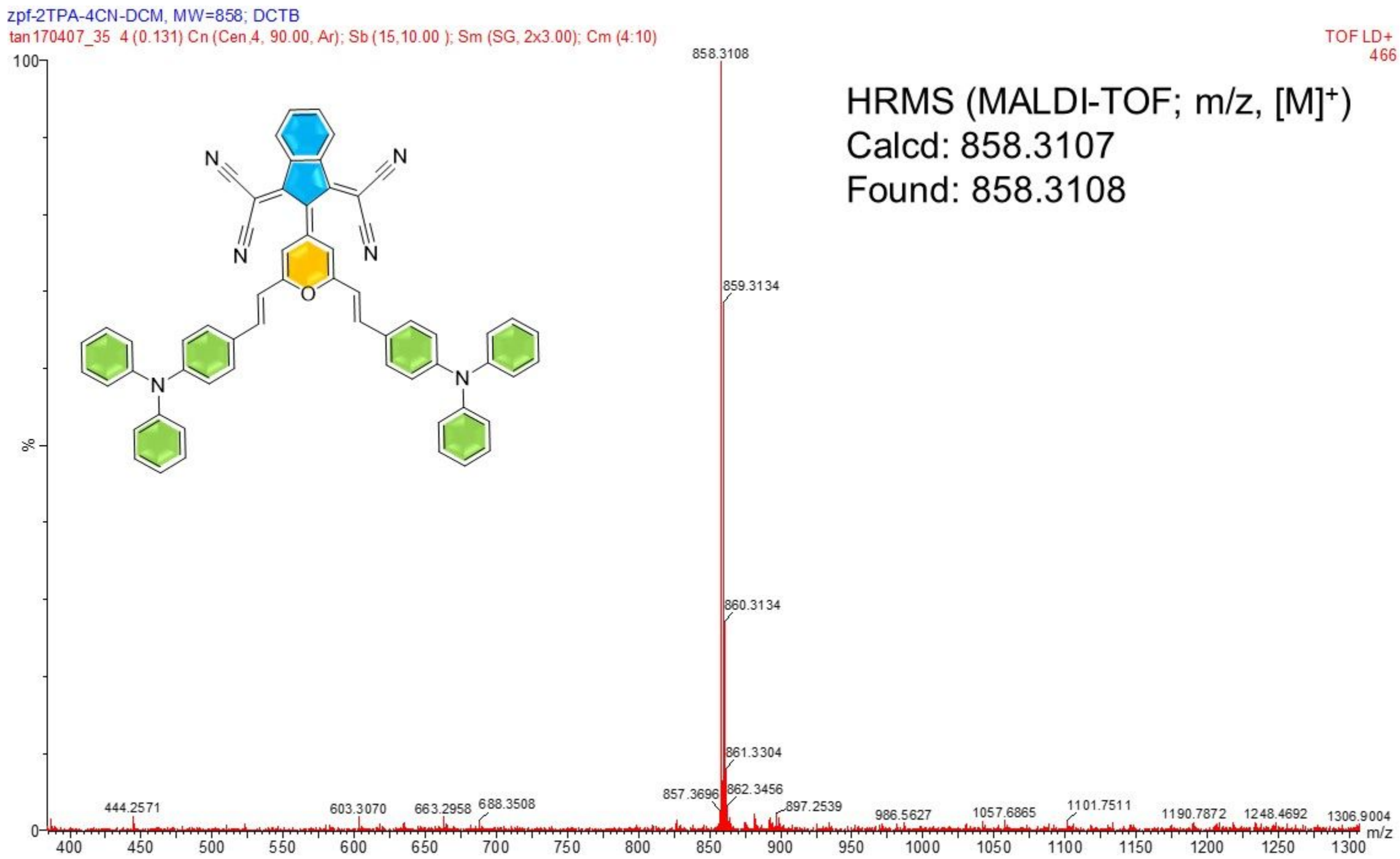

Figure S6. High resolution mass spectrum (MALDI-TOF) of TSPCI.

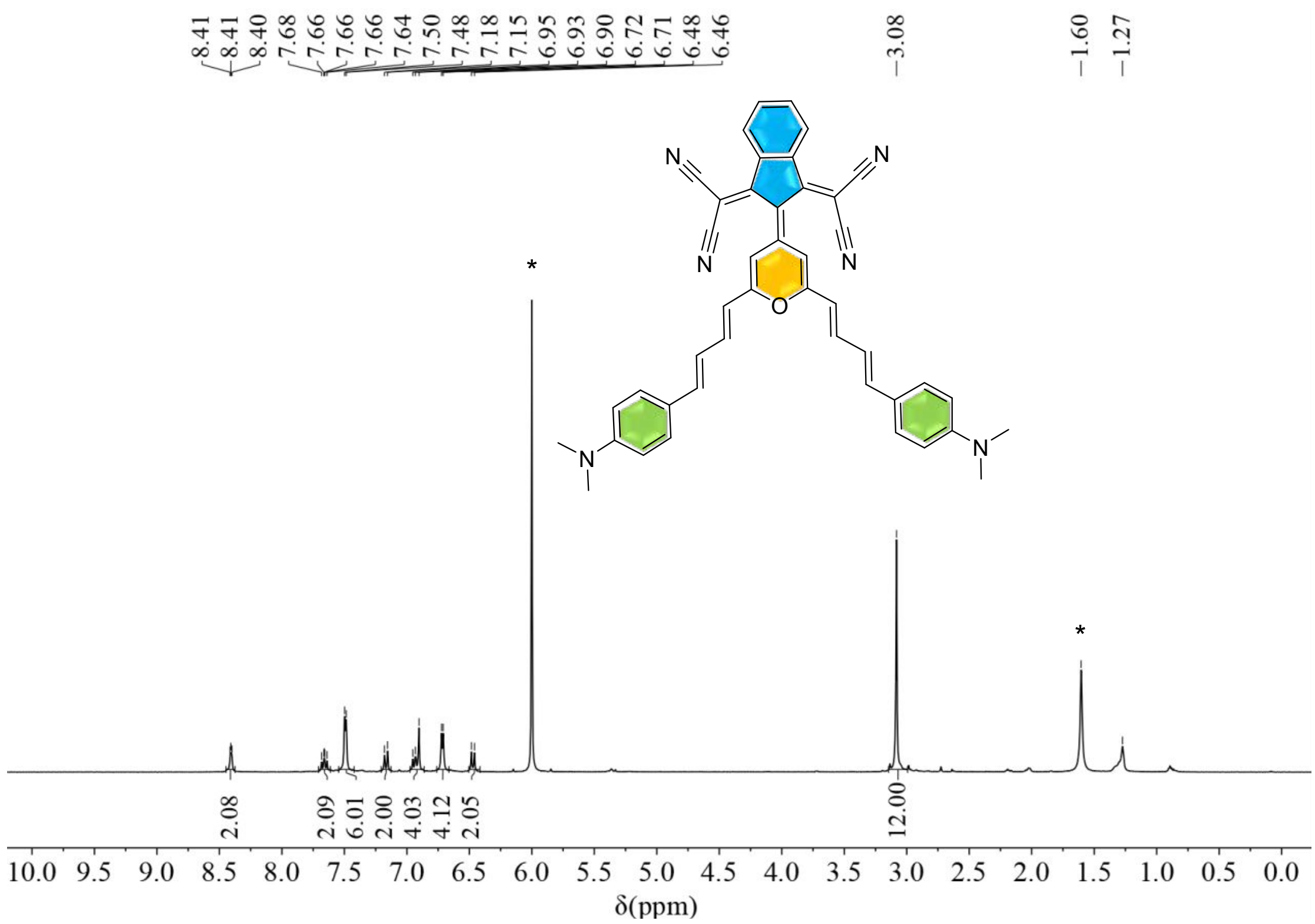

Figure S7. ^{1}H NMR spectrum of MASPCl in 1,2-dichloroethane-d_4.

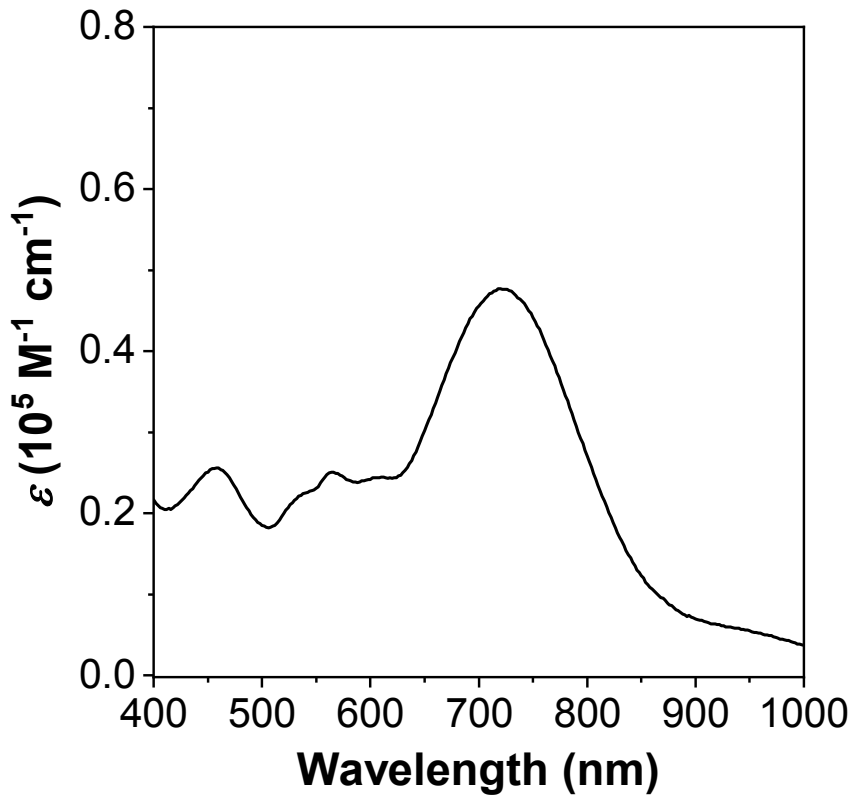

Figure S8. Molar extinction coefficient (ε) spectrum of MASPCI in THF.

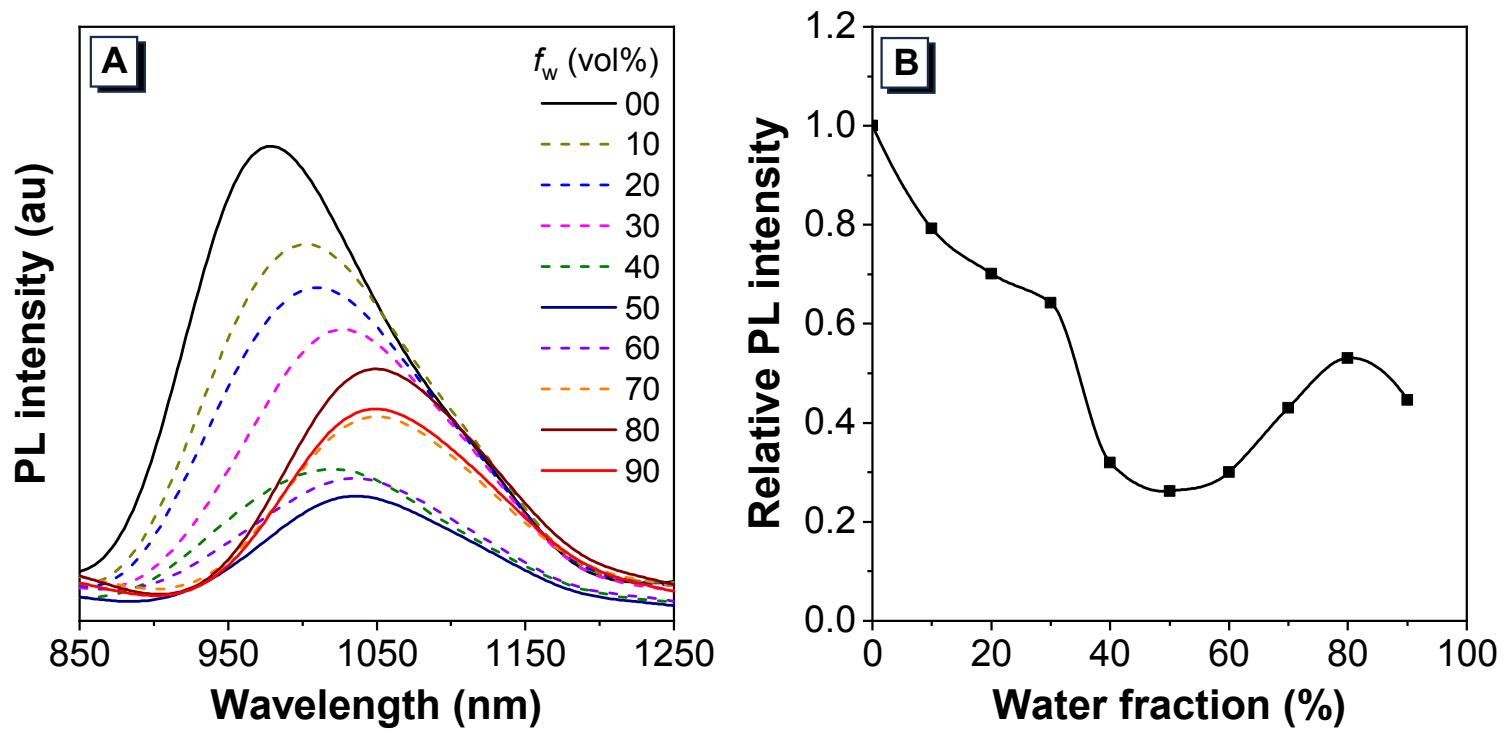

Figure S9. (A) PL spectra of MASPCI in THF and THF/water mixtures with the different f_w; (B) The plot of relative PL intensity at maximum emission wavelength versus the f_w. Concentration: 10 μM; excitation wavelength: 808 nm.

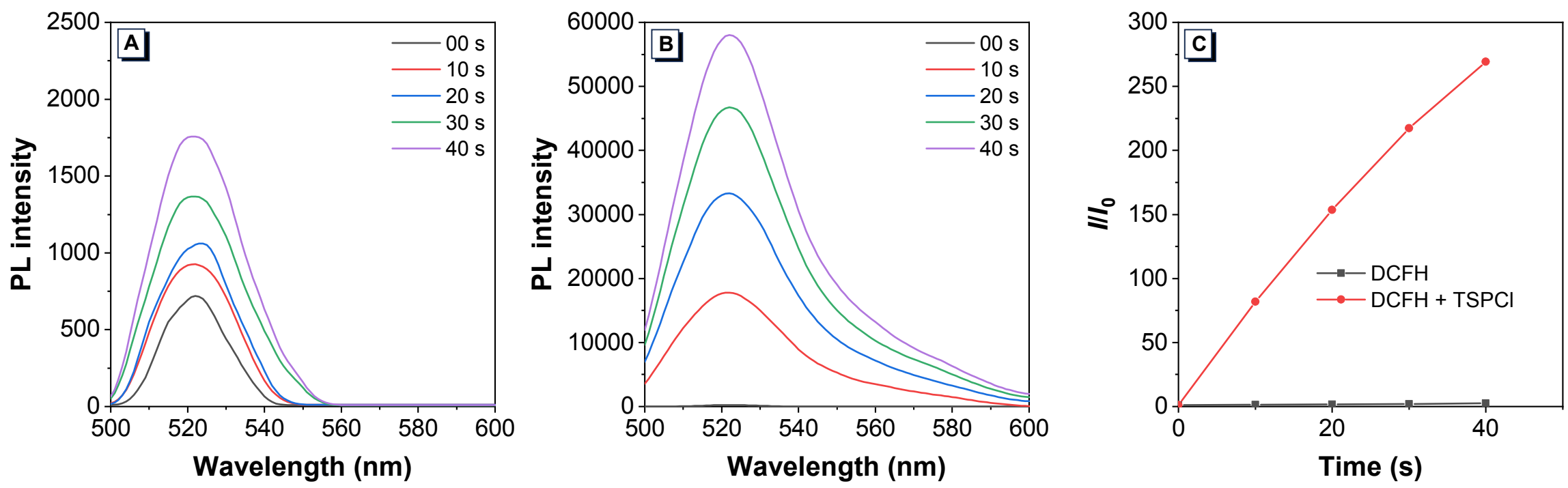

Figure S10. (A) DCFH and (B) DCFH + TSPCl with increasing irradiation time under an 808 nm laser (0.3 W cm^{-2}). (C) The PL intensity ratio (I/I_0) at 525 nm of DCFH upon 808 nm laser (0.3 W cm^{-2}) irradiation. Concentration: 10 μM.

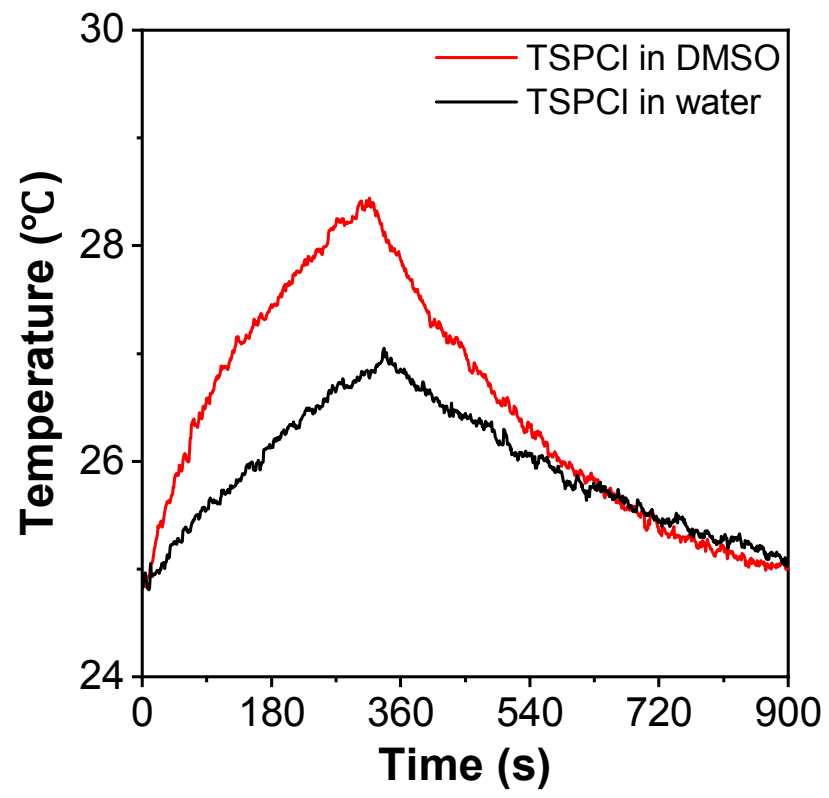

Figure S11. The photothermal response of the TSPCl (100 µM) in DMSO or water for 5 min with 808 nm laser irradiation (0.3 W cm^{-2}) and then the laser was shut off.

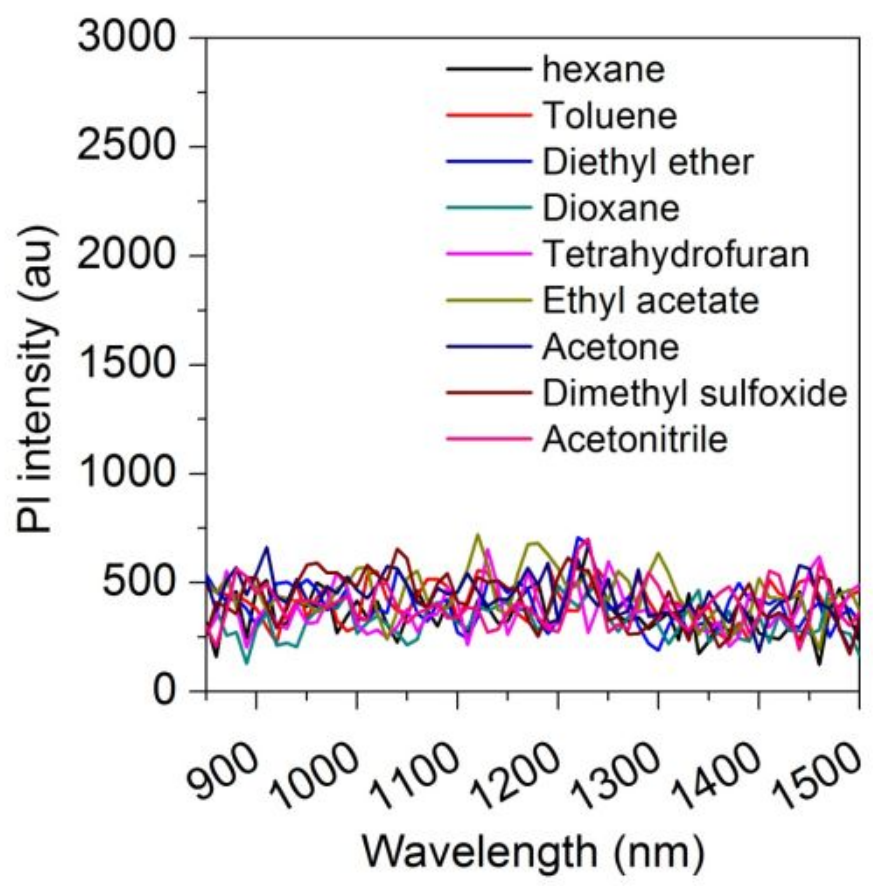

Figure S12. PL spectrum of TSPCI in solvents with different polarities.

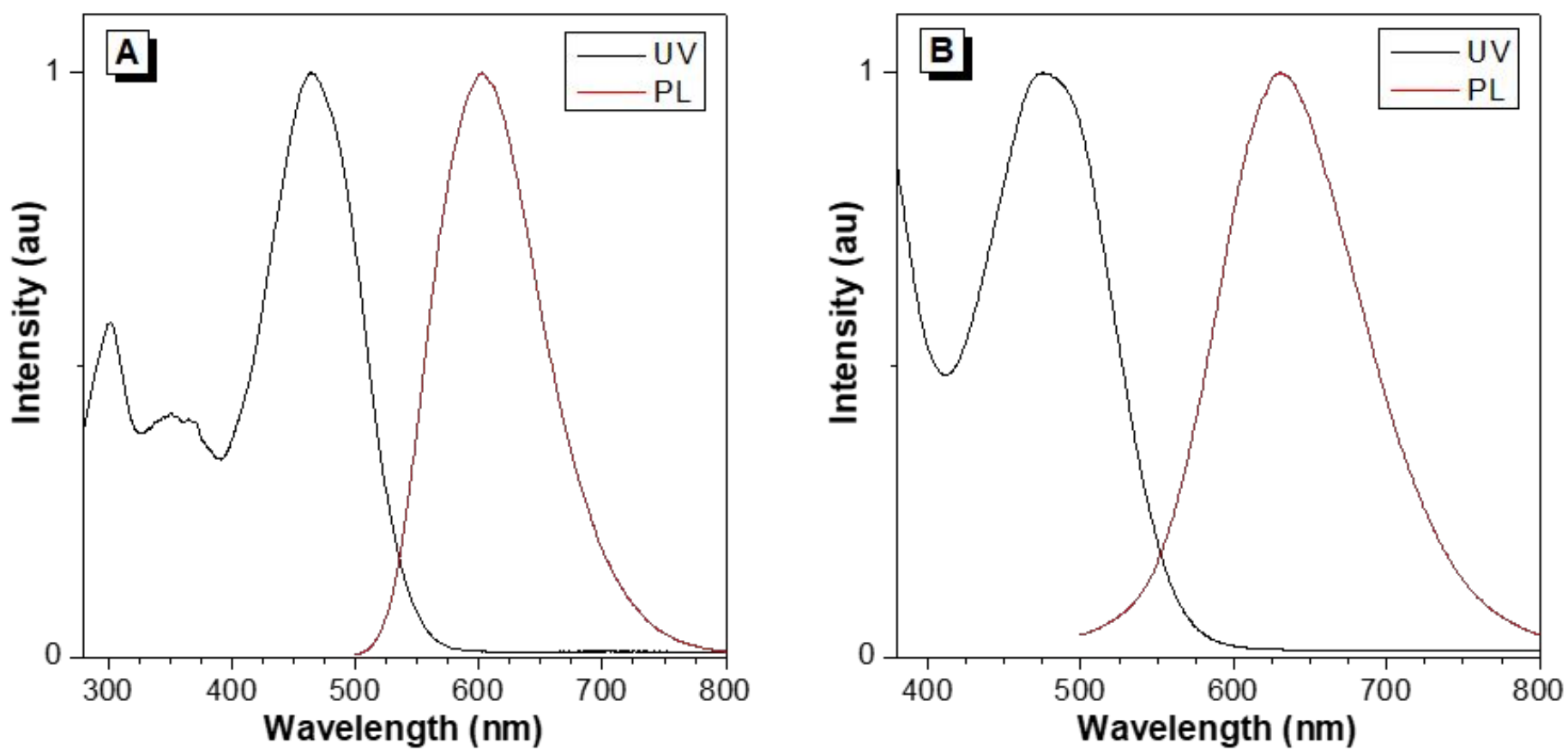

Figure S13. Normalized UV-vis and PL spectra of (A) TPA-DCM and (B) TTD in THF solution at a concentration of 10 µM.

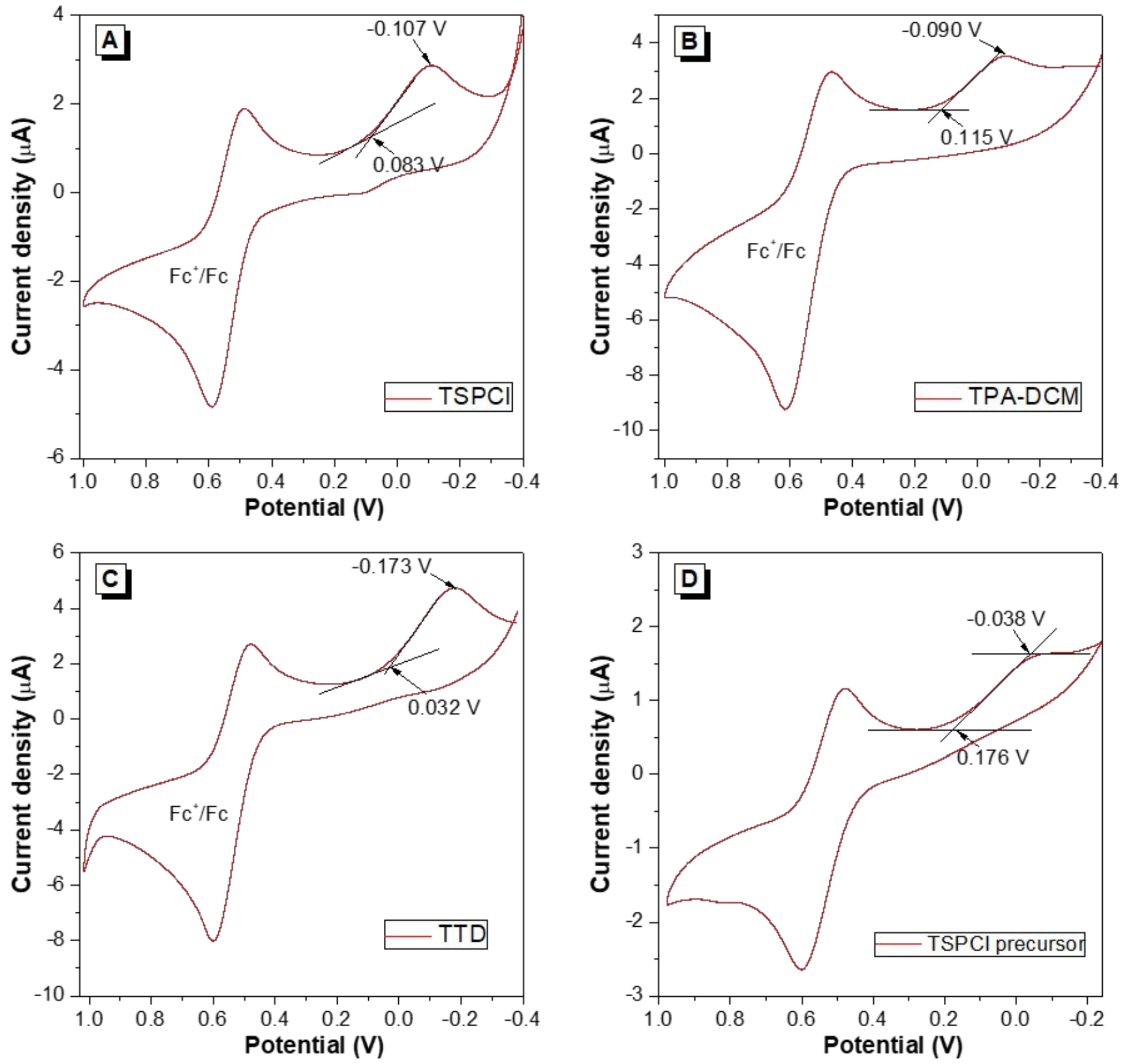

Figure S14. Cyclic voltammogram of (A) TSPCI, (B) TPA-DCM, (C) TTD and (D) SPCI at a concentration of 10 μM, quoted with reference to the internal ferrocene standard ($E_{1/2}$ = +540 mV vs. SCE). Scan rate: 100 mVs^{-1}; Electrolyte: $(n\text{-}C_4H_9)_4NPF_6$ (1 mM).

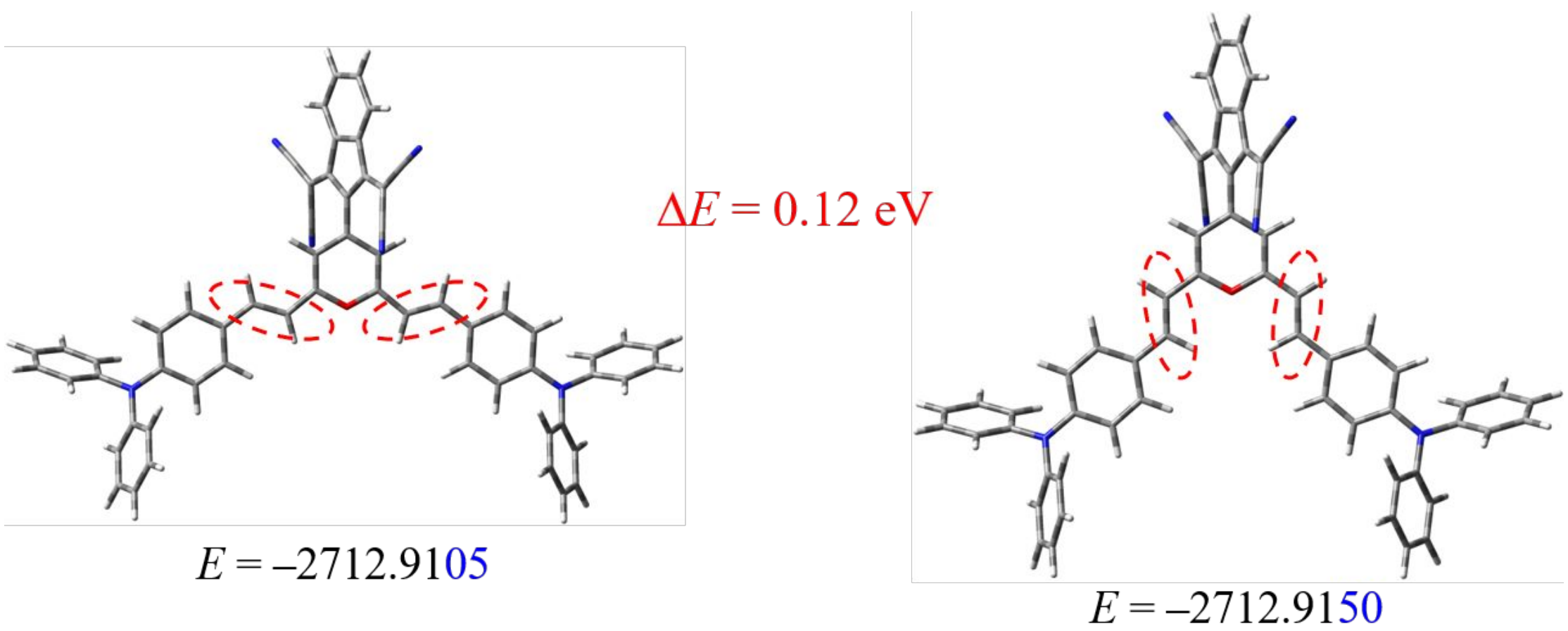

Figure S15. The optimized molecular geometries of TSPCI isomers.

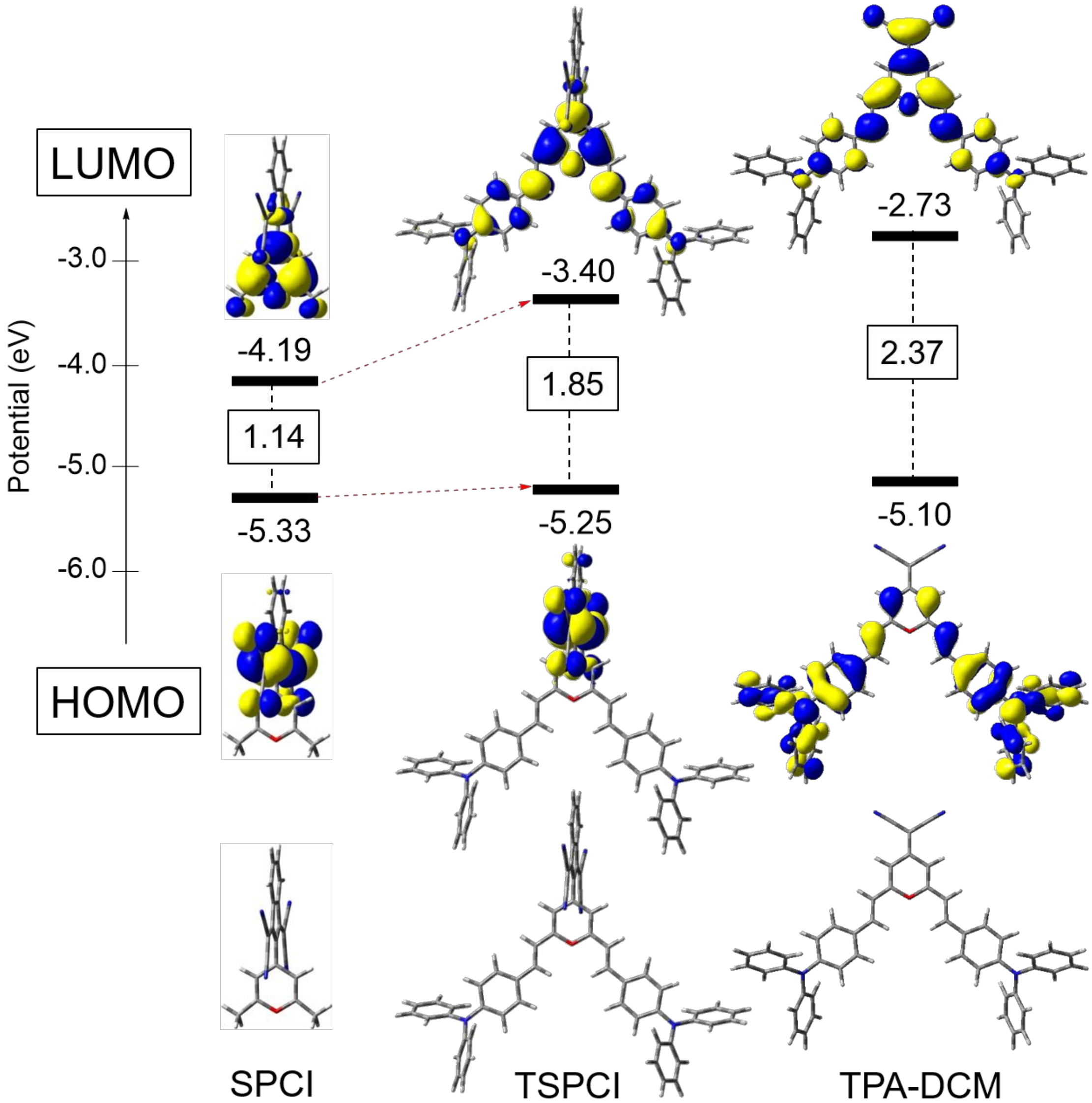

Figure S16. Molecular geometries, orbitals and energy levels of SPCI, TSPCI and TPA-DCM in S_1 state calculated with TD-DFT at the level of B3LYP/6-31G* based on solvation of THF, in which vertical excitation as linear response solvation was used.

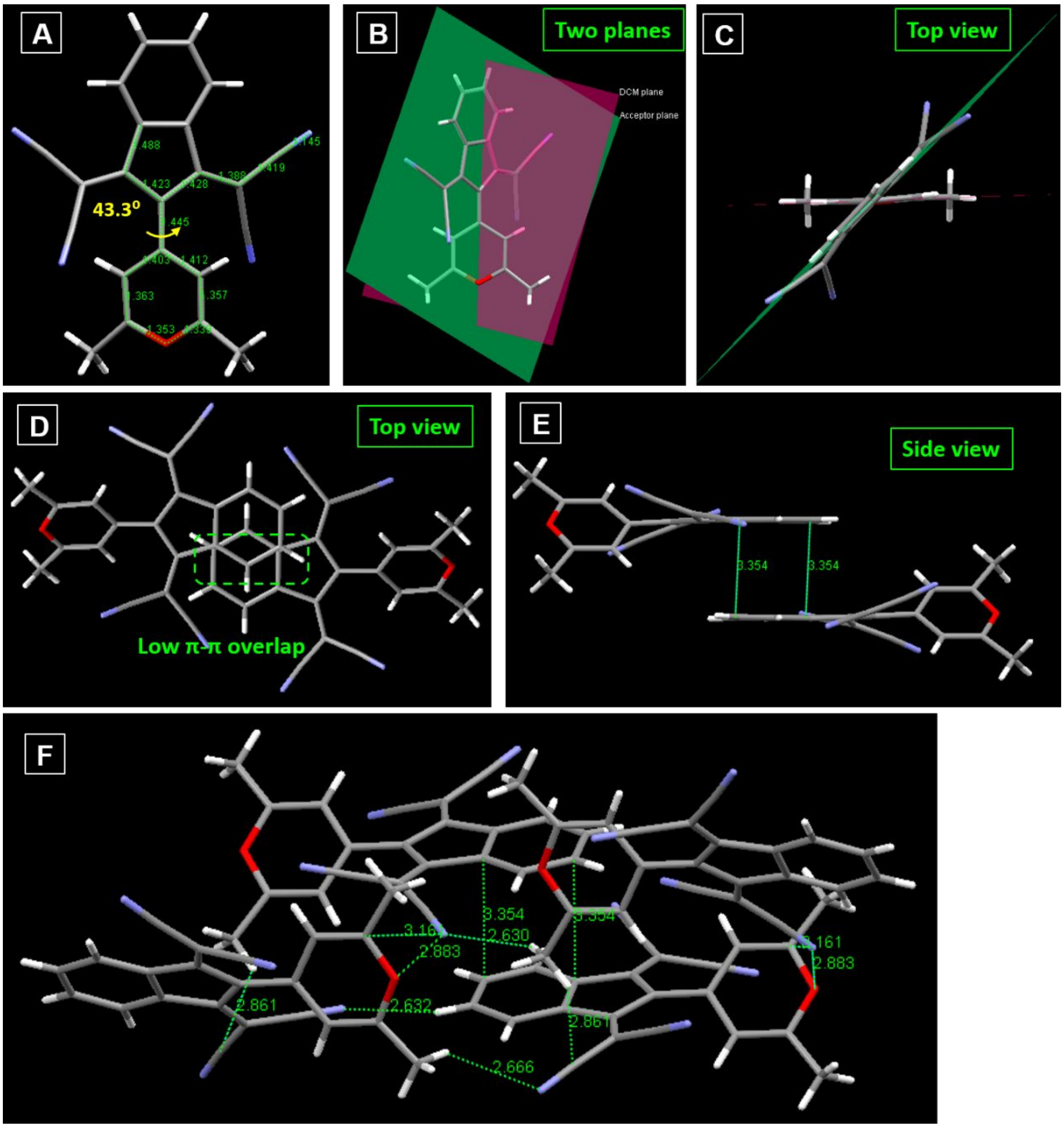

Figure S17. The packing structure of single crystals of SPCI.

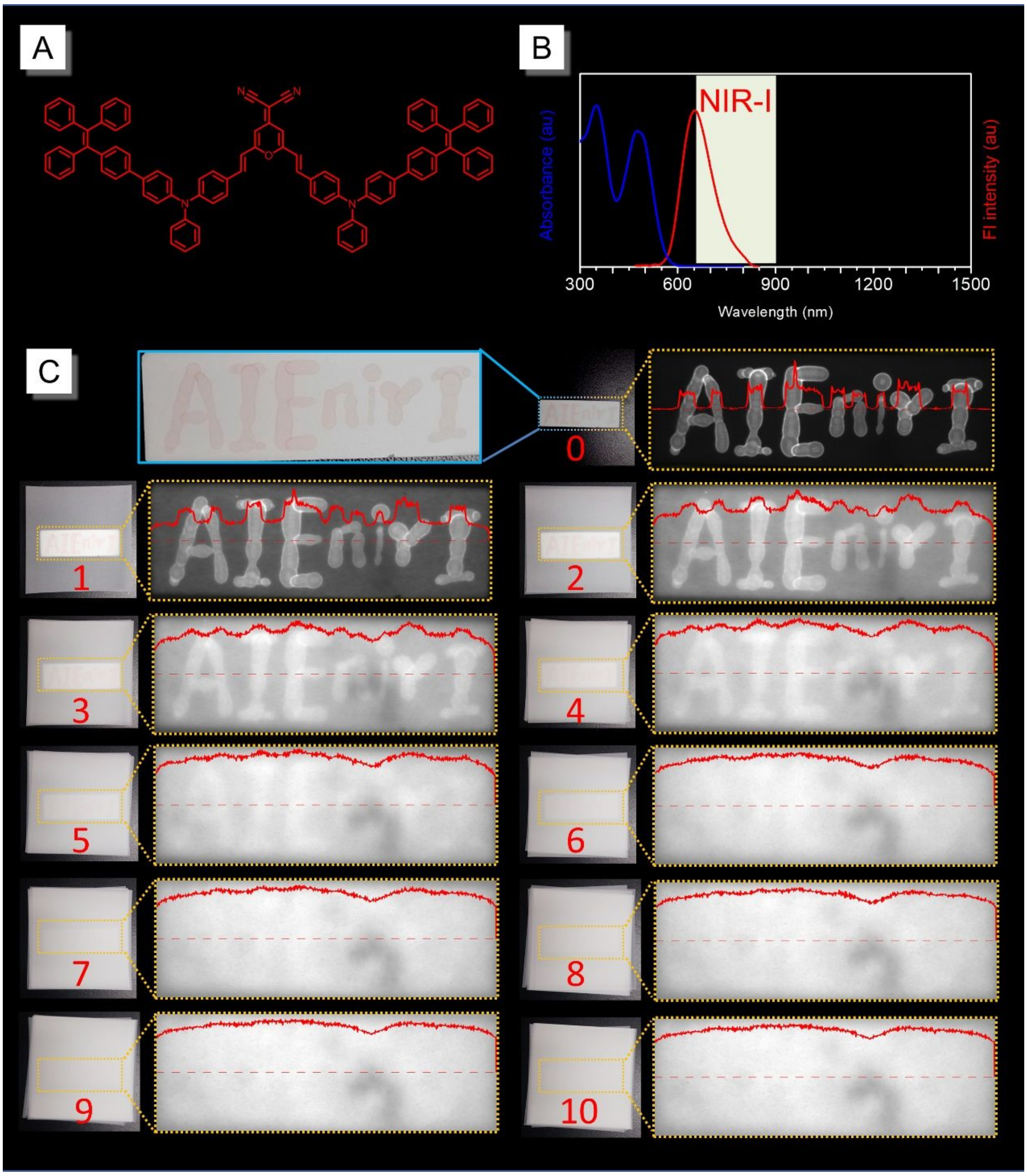

Figure S18. Spatial resolution analysis of NIR-I fluorescence imaging using TTD (NIR-I AIEgens). (A) The molecular structure of TTD. (B) The Normalized absorbance (blue) and photoluminescence (red) spectrum of TTD. (C) The photographs and NIR-I fluorescence images under different layer of papers covering the panel containing TTD (NIR-I AIEgens). Fluorescence intensity profiles (red) across a red dash line of interest with NIR-I emission.

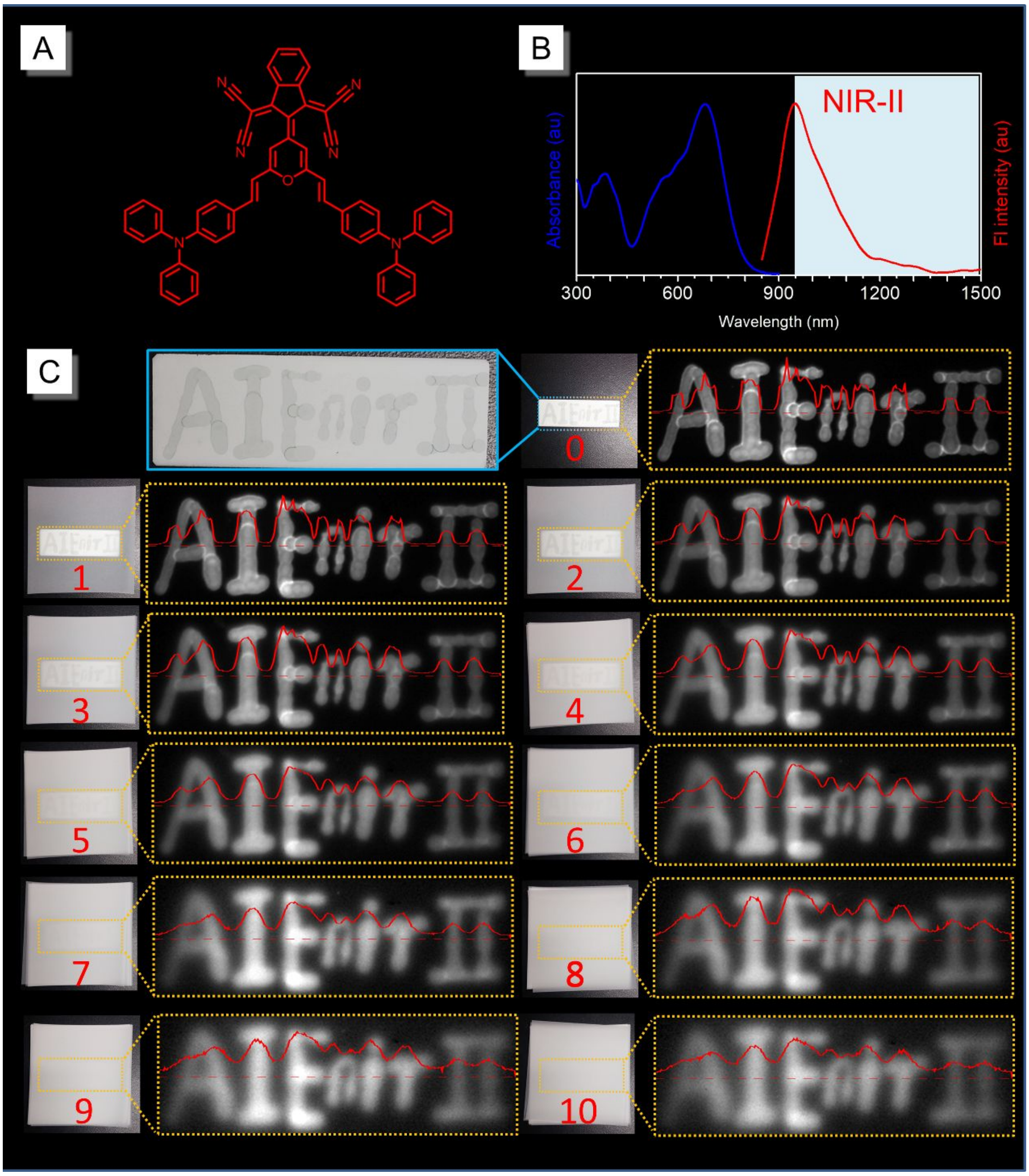

Figure S19. Spatial resolution analysis of NIR-II fluorescence imaging using TSPCI (NIR-II AIEgens). (A) The molecular structure of TSPCI. (B) The Normalized absorbance (blue) and photoluminescence (red) spectrum of TSPCI. (C) The photographs and NIR-II fluorescence images under different layer of papers covering the panel containing TSPCI (NIR-II AIEgens). Fluorescence intensity profiles (red) across a red dash line of interest with NIR-II emission.

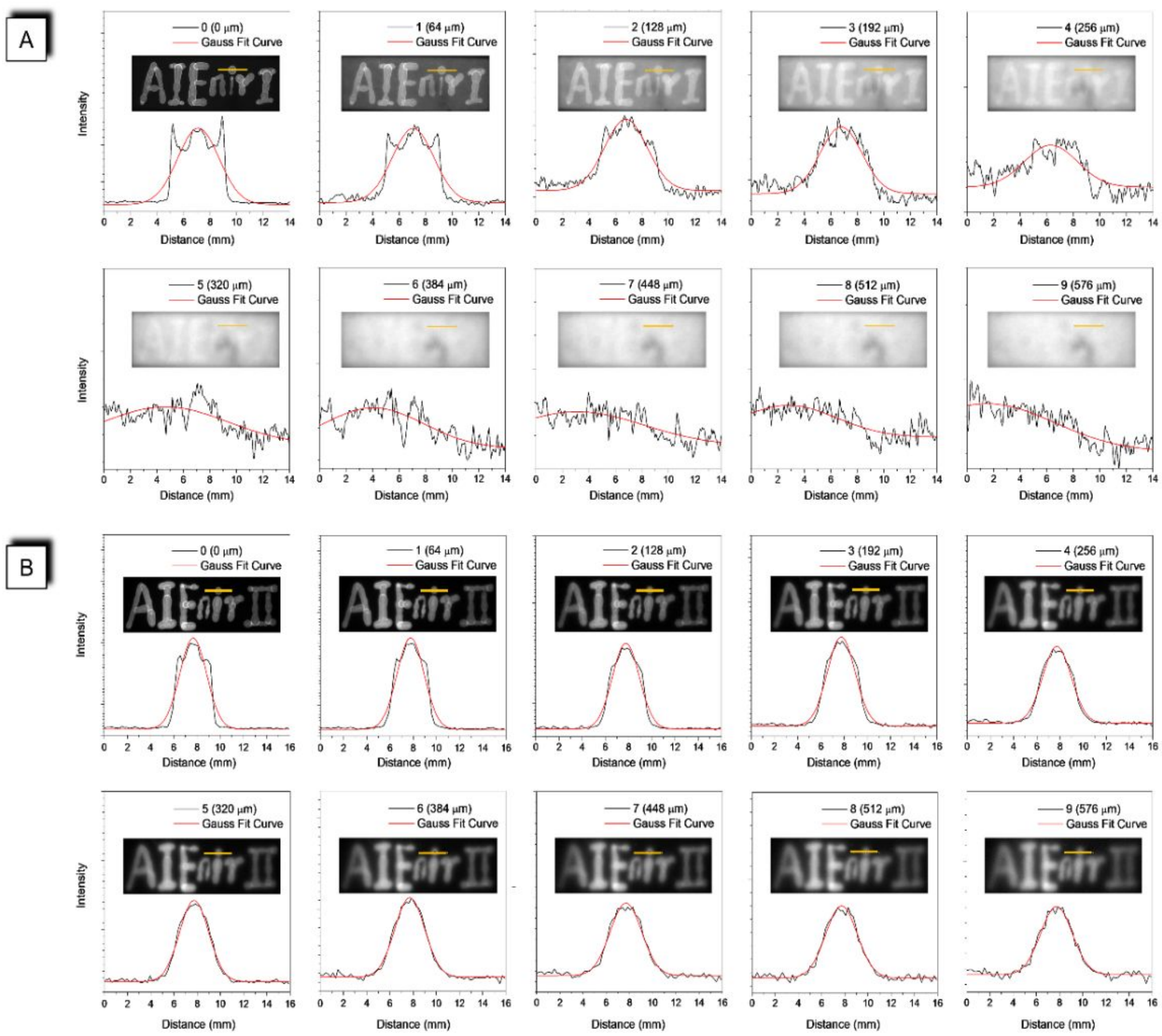

Figure S20. Full width at half maximum (FWHM) curves analysis of (A) TTD-drawn panel (NIR-I fluorescence) and (B) TSPCI-drawn panel (NIR-II fluorescence). The NIR fluorescence intensity profiles (black lines) and Gaussian fit (red lines) across the orange line of interest on the panels with letters drawn using TTD (A, NIR-I) and TSPCI (B, NIR-II) covered with different number of papers. Inset: Gray-scaled fluorescence images of the two panels with letters drawn using TTD (NIR-I) and TSPCI (NIR-II) covered with different number of papers (64 μm / paper) collected using Si-based VIS/NIR-I camera (A) and InGaAs-based NIR-II camera (B), respectively.

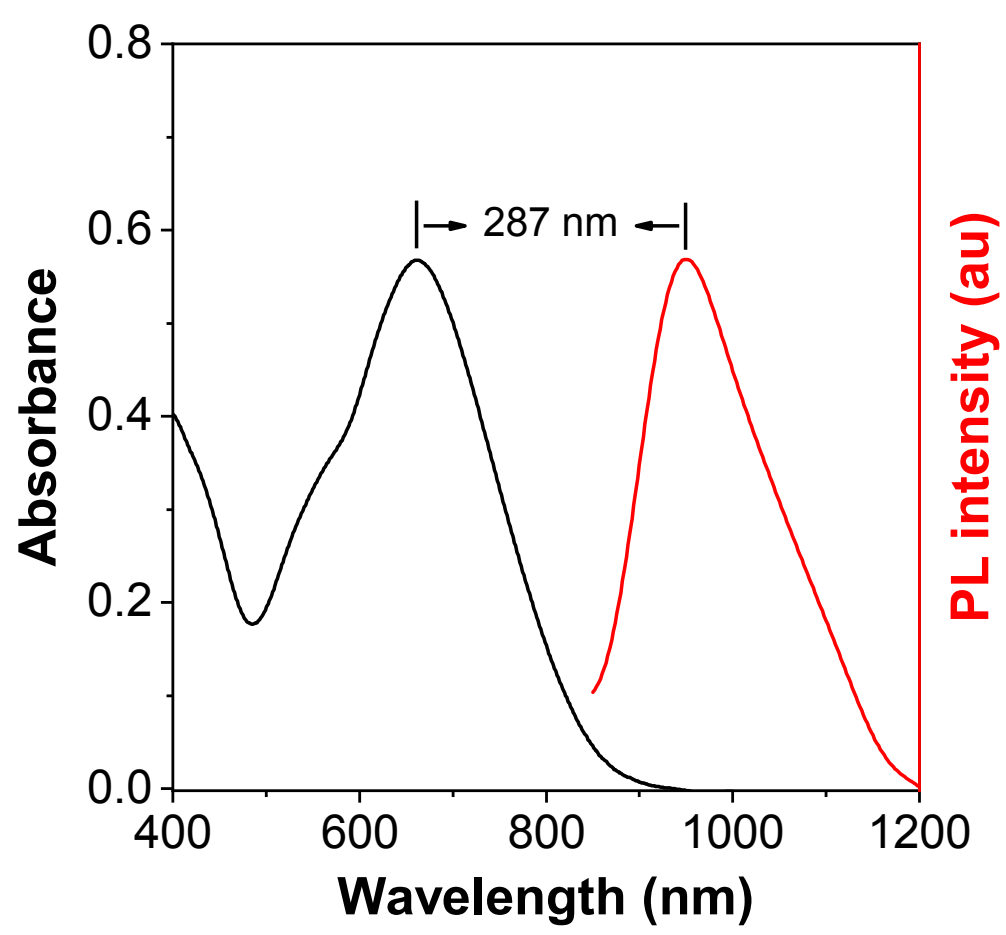

Figure S21. Absorption and fluorescence spectra of TSPCI in PBS.

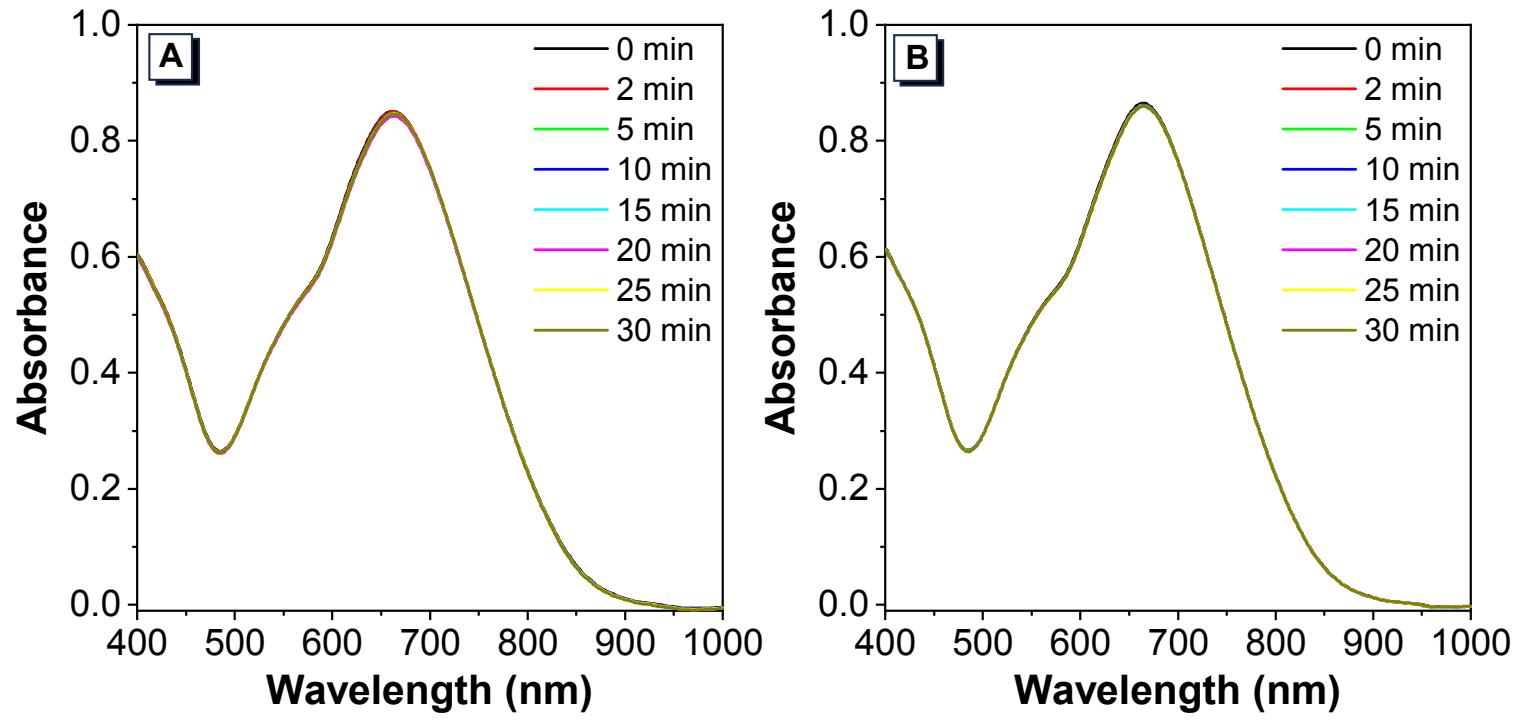

Figure S22. (A) Absorption spectra of TSPCl in aqueous solution under continuous 808 laser irradiation for different times. (B) Absorption spectra of TSPCl in 10% FBS solution under continuous 808 laser irradiation for different times.

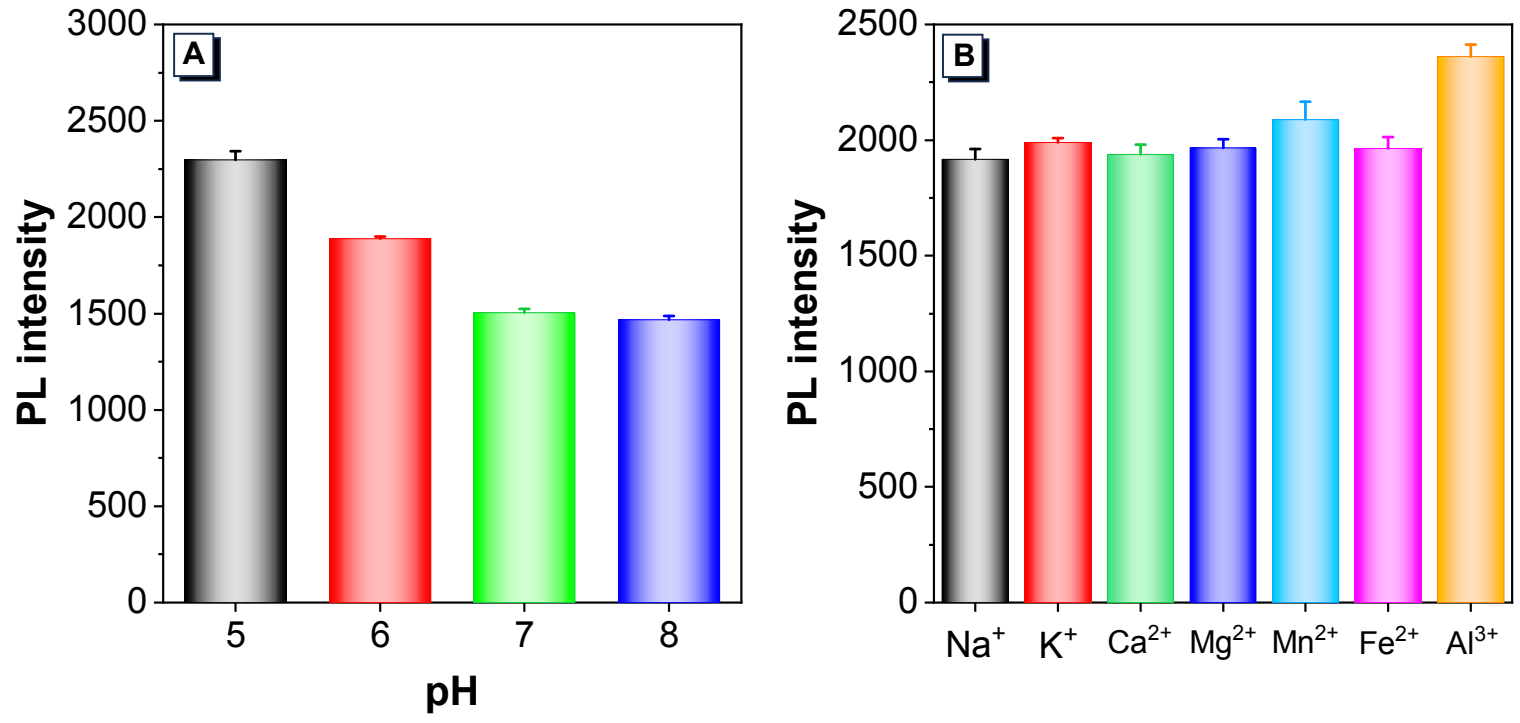

Figure S23. (A) PL intensity of TSPCI in DMSO at different pH values (pH 5.0–8.0). (B) PL intensity of TSPCI in the presence of various metal ions (Na^+, K^+, Ca^{2+}, Mg^{2+}, Mn^{2+}, Fe^{2+}, Al^{3+}). Ionic concentration: 100 mM.

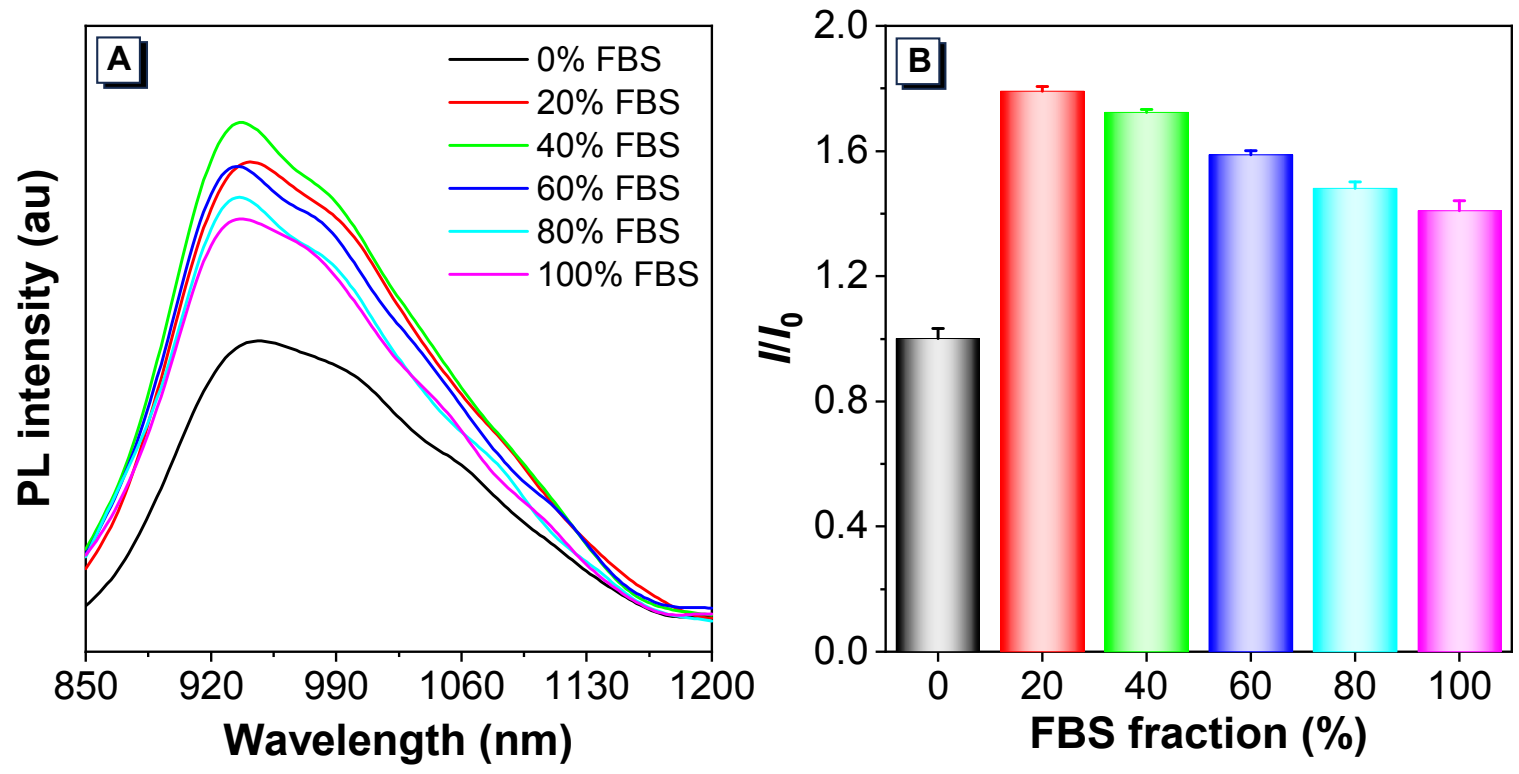

Figure S24. (A) Fluorescence spectra of TSPCI in water/PBS mixtures containing different volume fractions of fetal bovine serum (FBS, 0%–100%). (B) Plot of fluorescence intensity versus FBS fraction at the maximum emission wavelength. Concentration: 10 μM; λ_{ex} = 808 nm laser.

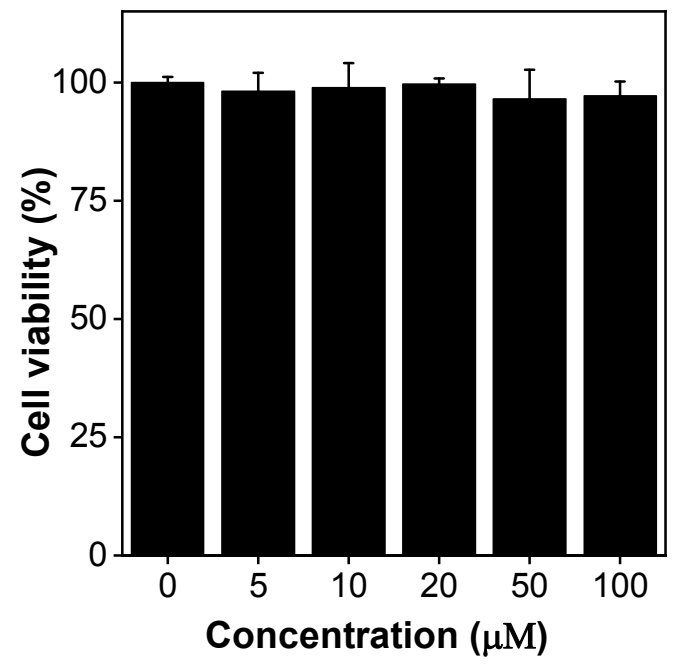

Figure S25. Cell viability of NIH-3T3 cells after incubation with different concentrations of TSPCI (0–100 μM) for 24 h, determined by MTT assay. Data are presented as mean ± SD (n = 3).

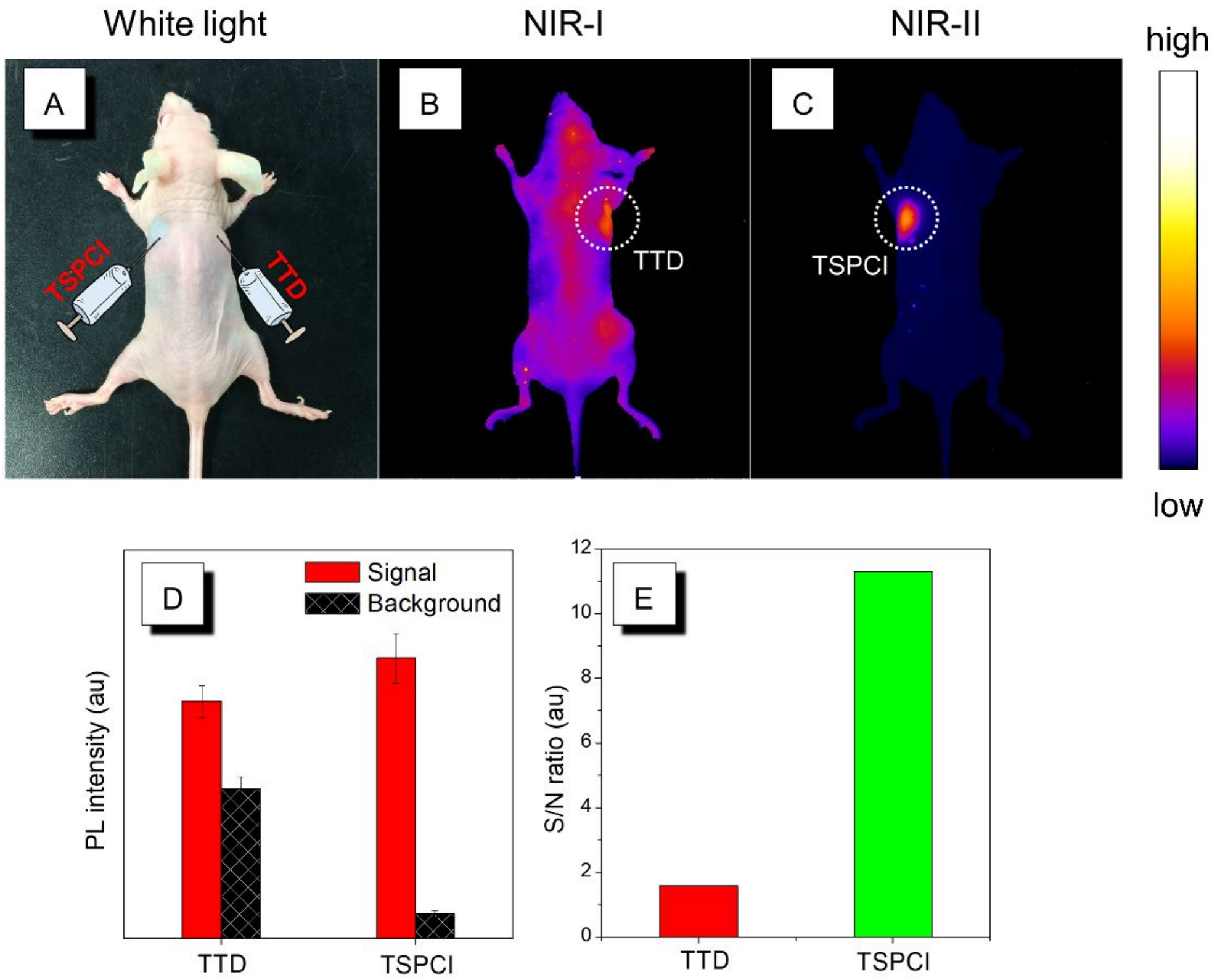

Figure S26. In vitro comparison of NIR-I and NIR-II fluorescence imaging of TTD and TSPCI. The photograph (A), NIR-I image (B) and NIR-II image (C) of the nude mouse with pre-injection TSPCI (NIR-II AIEgens, left side) and TTD (NIR-I AIEgens, right side). (D) The signal of the injection site and the background signal from the mouse body in(B) and (C). (E) The corresponding signal background ratio (S/N ratio) analysis of the white dot line circle area in (B) and (C) Autofluorescent signals from the mouse body in (C) are significantly lower than that in (B).

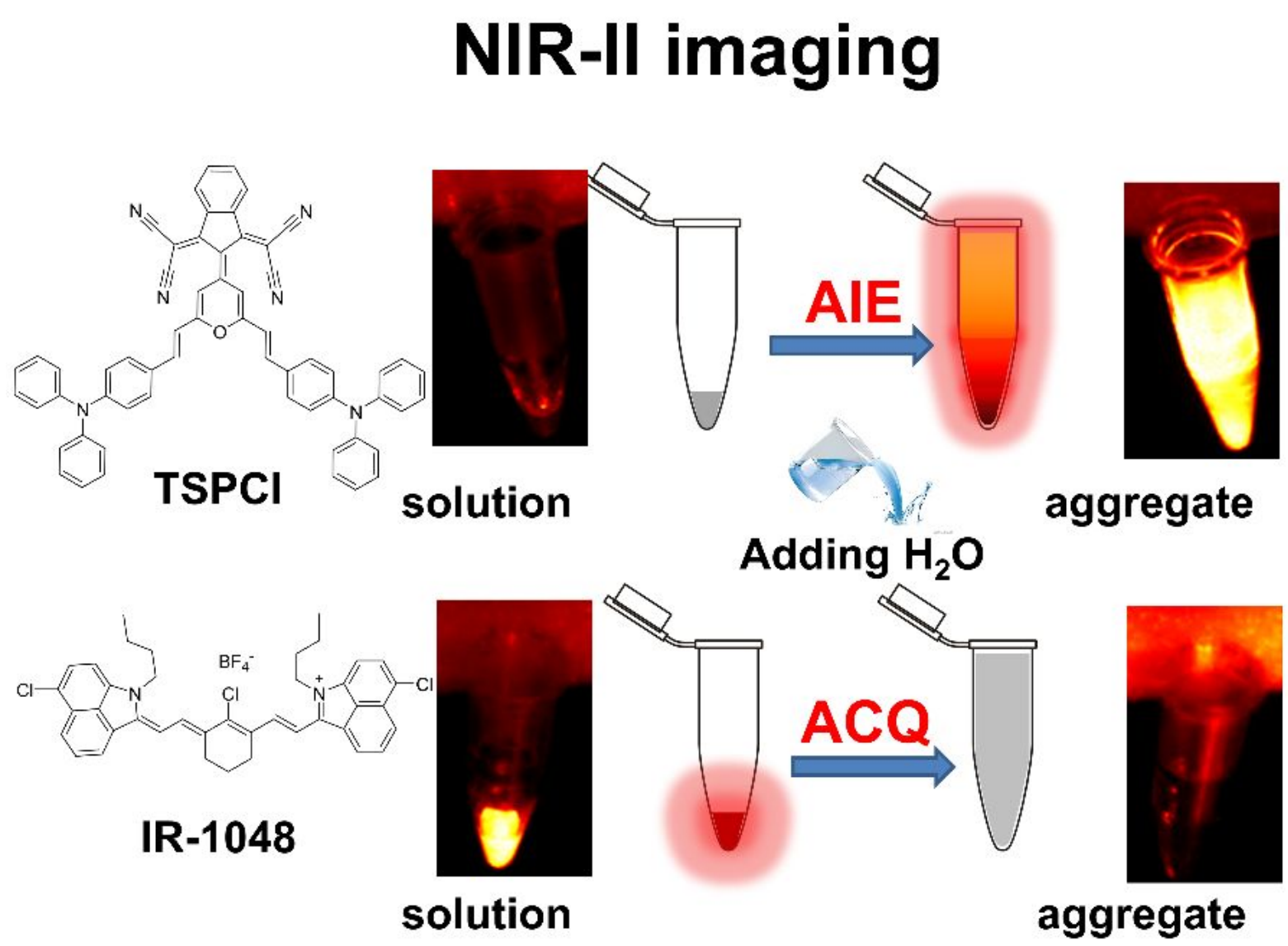

Figure S27. The NIR-II fluorescence imaging of TSPCI and IR-1048 before and after addition of water (poor solvent) into their dilute solution (good solvent).

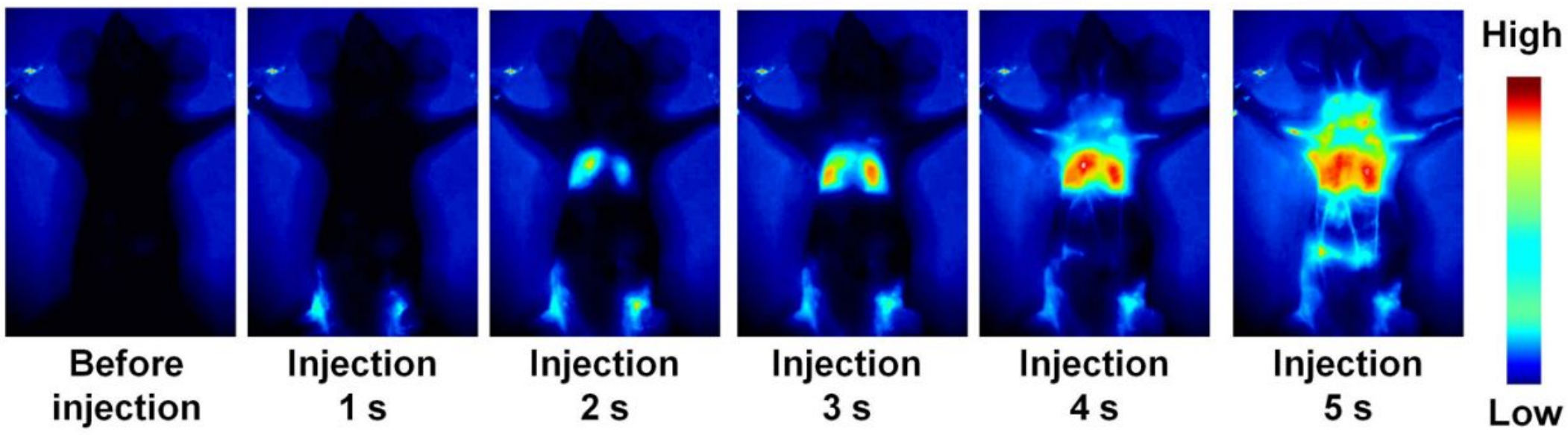

Figure S28. Time course NIR-II fluorescence images of the nude mouse before and after intravenous injection of TSPCI solution.

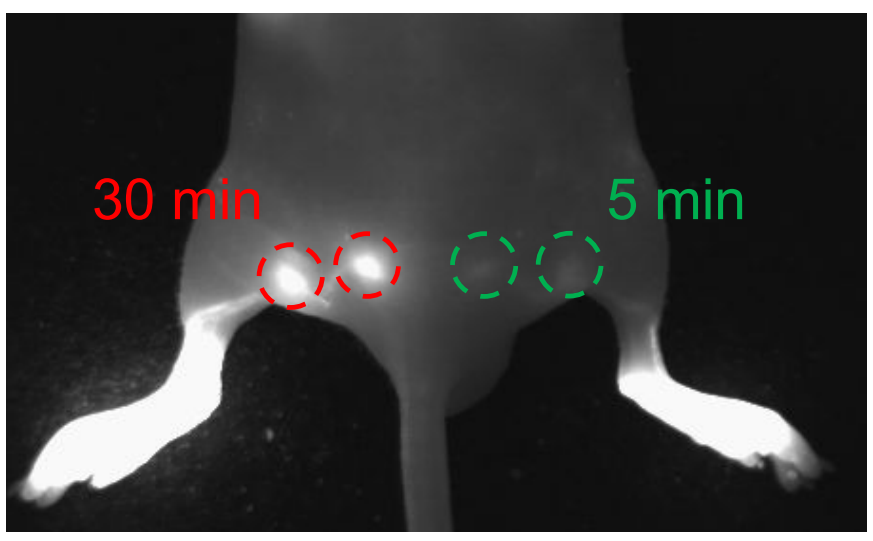

Figure S29. NIR-II imaging of the lymph nodes in mice after injection of TSPCl for 30 min (left) and 5 min (right).

Table S1. Photophysical preparties of TSPCI, TPA-DCM and TTD.[a)]

	λ_{abs} (nm)	λ_{em} (nm)	E_g (V)[c)]	E_{ox} (V)[d)]	HOMO (V)[e)]	LUMO (V)[e)]
TSPCI	681	nd[b)]	1.57	–0.01	–5.09	–3.52
TPA-DCM	465	602	2.27	0.01	–5.11	–2.84
TTD	476	631	2.18	–0.01	–5.03	–2.85

[a)] Recorded in THF solutions at 298 K. [b)] nd: no detected. [c)] The band gap (E_g) calculated from the onset of UV-vis absorption spectrum. [d)] Recorded in THF. Scan rate: 100 mVs^{-1}; Electrolyte: (n-$C_4H_9)_4NPF_6$; Potentials are quoted with reference to the internal ferrocene standard ($E_{1/2}$ = +540 mV vs. SCE). [e)] HOMO and LUMO energies are calculated using formula HOMO = −[5.1 + E_{ox}], LUMO = HOMO + E_g.

Table S2. Calculation data of TSPCI in S_0 state.[a)]

Excited states	Configurations	E (eV)	λ (nm)	f_{os}
S_{01}	H → L (99%)	1.72	719.03	0.91
S_{02}	H–2 → L (11%), H–1 → L (89%)	1.80	688.19	0.65
S_{03}	H → L+1 (99%)	2.06	601.84	0.00

[a)] Calculated with TD-DFT at the level of B3LYP/6-31G* based on solvation of THF, in which the vertical excitation as linear response solvation was used. S_{01}, S_{02} and S_{03} denoted the first and second vertical transition from S_0 state to S_1, S_2 and S_3 states, respectively, and f_{os} denoted oscillator strength between the ground and excited states.

Table S3. Calculation data of TSPCI in S_1 state.[a)]

Excited states	Configurations	E (eV)	λ (nm)	f_{os}
S_{10}	H → L (99%)	1.21	1020.96	0.00

[a)] Calculated with TD-DFT at the level of B3LYP/6-31G* based on solvation of THF, in which the vertical excitation as linear response solvation was used. S_{10} denoted the first vertical radiation from the S_1 state to the S_0 state, and f_{os} denoted oscillator strength between the ground and excited states.

Table S4. The specific dihedral angles of TSPCI in S_0 and S_1 states.[a)]

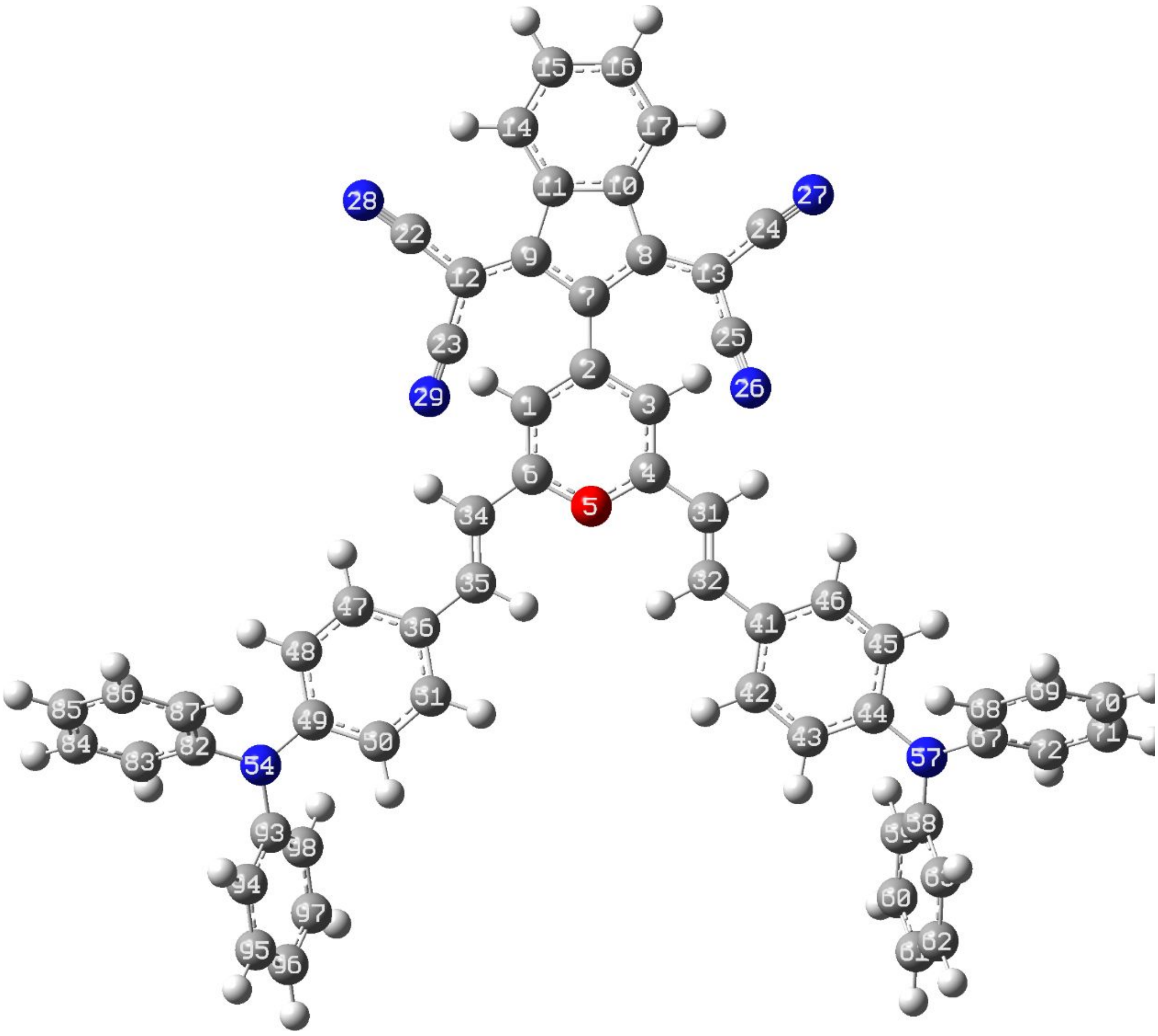

State	S_0	S_1
C11–C9–C12–C22	–12.49	0.002
C7–C8–C13–C25	–9.06	0.004
C1–C2–C7–C8	–54.99	–90.07
C3–C2–C7–C9	–55.02	–89.93
O5–C6–C34–C35	0.80	0.13
O5–C4–C31–C32	1.09	–0.14
C34–C35–C36–C47	1.40	1.15
C31–C32–C41–C46	-0.33	–1.13
C48–C49–N54–C82	–27.19	–36.13
C50–C49–N54–C93	–26.45	–35.50
C45–C44–N57–C67	27.15	36.14
C43–C44–N57–C58	26.45	35.51

[a)] Calculated with TD-DFT at the level of B3LYP/6-31G* based on solvation of THF. The unit is degree.

Table S5. The specific bond lengths of TSPCI in S_0 and S_1 states.[a)]

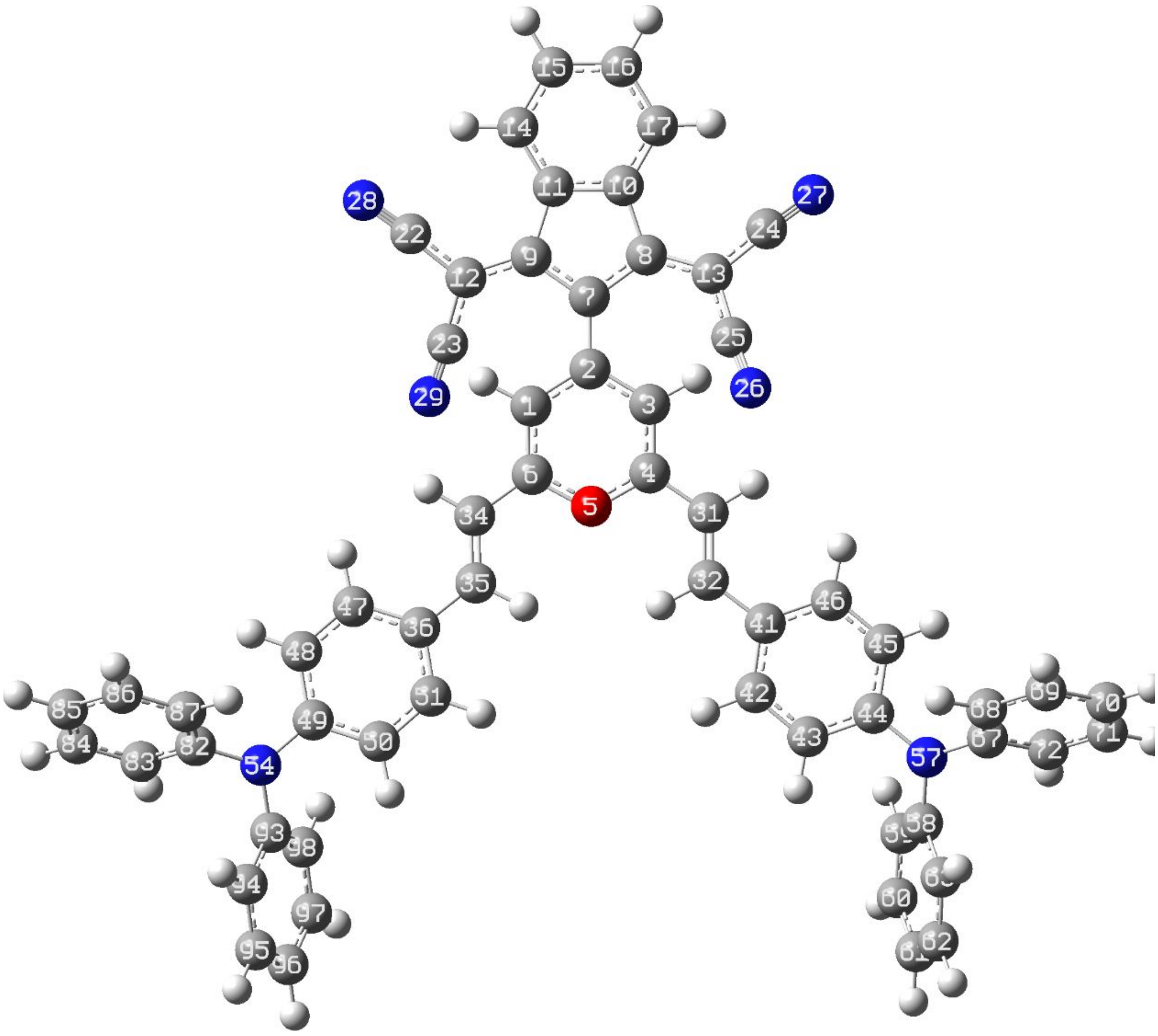

State	S_0	S_1
C2-C7	1.463	1.475 (↑0.012)
C7–C8	1.430	1.435 (↑0.005)
C8–C10	1.484	1.480 (↑0.004)
C10–C11	1.416	1.416
C8–C13	1.403	1.393 (↓0.010)
C13–C24	1.423	1.426 (↑0.003)
C24–N27	1.168	1.165 (↓0.003)
C1–C2	1.408	1.411
C1–C6	1.384	1.379 (↓0.005)
O5–C6	1.360	1.381 (↑0.021)
C6–C34	1.429	1.429
C34–C35	1.363	1.362 (↓0.001)

a) Calculated with TD-DFT at the level of B3LYP/6-31G* based on solvation of THF. The unit is Å.

Table S6. The specific dihedral angles and bond lengths of SPCI single-crystal and optimized structures in S_0 and S_1 states.[a)]

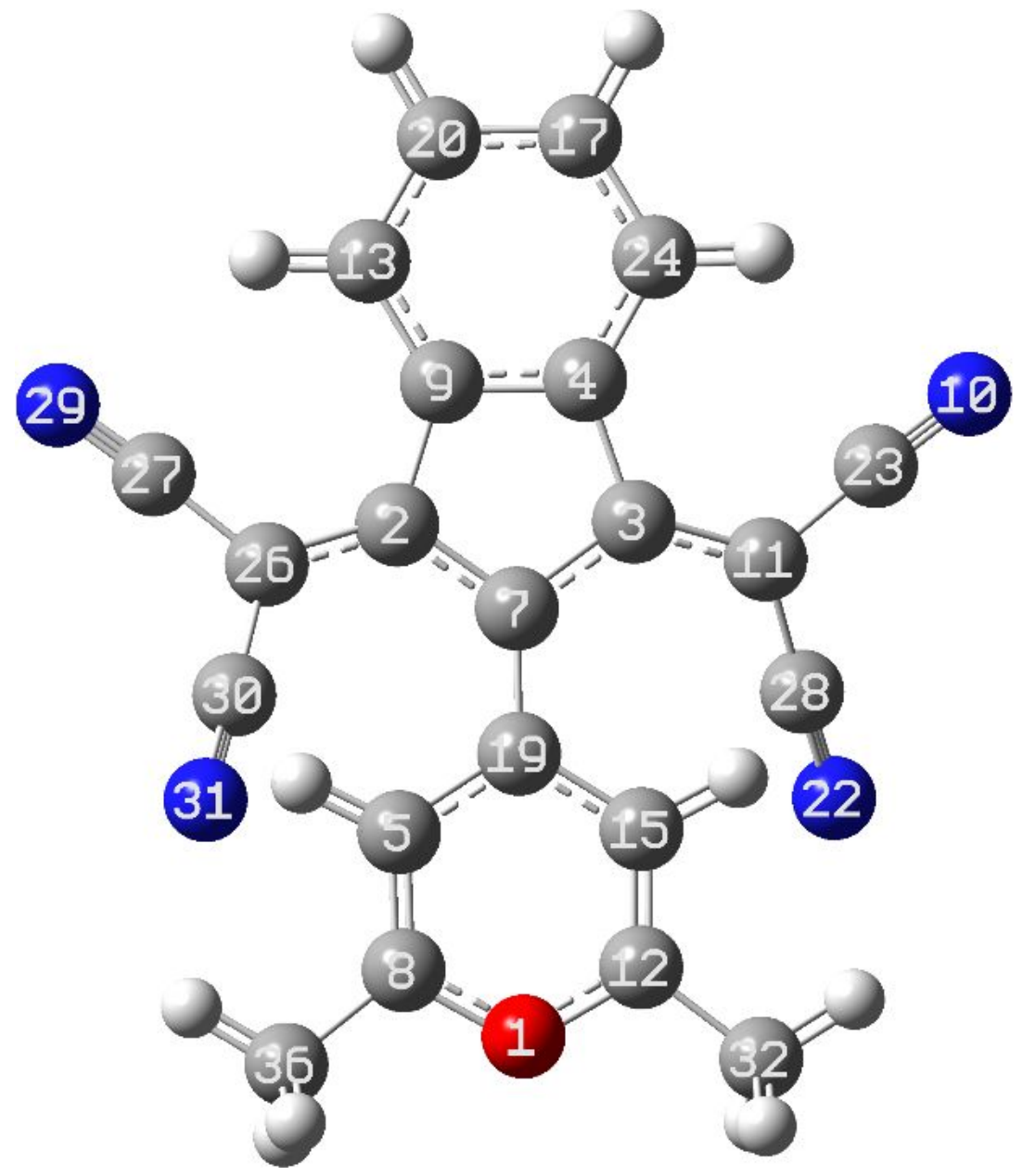

State	Single-crystal	S_0	S_1
C4–C3–C11–C23	17.92	14.798	0.002
C7–C3–C11–C28	14.06	8.978	0.013
C3–C7–C19–C15	43.33	43.424	89.998
C9–C2–C26–C27	19.31	14.837	–0.001
C7–C2–C26–C30	13.30	9.000	–0.013
C2–C7–C19–C5	42.33	43.42	90.003
C4–C9	1.405	1.417	1.416 (↓0.001)
C4–C3	1.482	1.479	1.483 (↑0.004)
C3–C11	1.388	1.397	1.397
C11–C23	1.419	1.427	1.425 (↓0.002)
C23–N10	1.145	1.166	1.166
C3–C7	1.428	1.447	1.431 (↓0.016)
C7–C19	1.445	1.433	1.485 (↑0.052)
C19–C5	1.412	1.425	1.418 (↓0.007)
C5–C8	1.357	1.362	1.362
C8–O1	1.339	1.356	1.381 (↑0.025)

[a)] Calculated with TD-DFT at the level of B3LYP/6-31G* based on solvation of THF. The unit of dihedral angle and bond length are degree and Å, respectively.

Table S7. Crystal data and structure refinement for SPCI.

Formula weight	348.36
Temperature/K	297.0
Crystal system	monoclinic
Space group	$P2_1/n$
a /Å	8.0272(4)
b /Å	12.3563(5)
c /Å	17.9575(7)
α /°	90
β /°	96.973
γ /°	90
Volume /$Å^3$	1767.97
Z	4
ρ_{calc} /gcm^{-3}	1.309
μ /mm^{-1}	0.0941

Table S8. Blood biochemistry test regarding liver and kidney function of the mice after different treatments (n = 3 independent samples per group).

	ALT (U/L)	AST (U/L)	ALB (g/L)	BUN (mmol/L)	CREA (μmol/L)	UA (μmol/L)
PBS	25.42 ± 1.53	155.45 ± 4.25	32.64 ± 3.52	14.89 ± 3.15	8.85 ± 1.37	18.25 ± 3.18
TSPCl	24.29 ± 1.19	153.27 ± 10.58	32.98 ± 5.58	13.58 ± 3.75	8.16 ± 0.95	17.65 ± 2.29

References:

1. Wang, X.; Guo, Z.; Zhu, S.; Liu, Y.; Shi, P.; Tian, H.; Zhu, W.-H., Rational design of novel near-infrared fluorescent DCM derivatives and their application in bioimaging. *J. Mater. Chem. B* **2016,** *4*, 4683-4689.
